# Mitogen activated protein kinase phosphatase 5 alleviates liver ischemia–reperfusion injury by inhibiting TAK1/JNK/p38 pathway

**DOI:** 10.1038/s41598-023-37768-9

**Published:** 2023-07-10

**Authors:** Qiwen Yu, Sanyang Chen, Jiye Li, Hongwei Tang, Jihua Shi, Wenzhi Guo, Shuijun Zhang

**Affiliations:** 1 Department of Hepatobiliary and Pancreatic Surgery, the First Affiliated Hospital of Zhengzhou University, 1 Jianshe East Road, Erqi, Zhengzhou, Henan China; 2 Henan Key Laboratory of Digestive Organ Transplantation, Zhengzhou, Henan China; 3 Department of Emergency Surgery, the First Affiliated Hospital of Zhengzhou, Zhengzhou, Henan China

**Keywords:** Liver diseases, Inflammasome

## Abstract

Mitogen activated protein kinase phosphatase 5 (MKP5) is a member of the MKP family and has been implicated in diverse biological and pathological conditions. However, it is unknown what role MKP5 plays in liver ischemia/reperfusion (I/R) injury. In the present study, we used MKP5 global knockout (KO) and MKP5 overexpressing mice to establish a liver I/R injury model in vivo, and MKP5 knockdown or MKP5 overexpressing HepG2 cells to establish a hypoxia-reoxygenation (H/R) model in vitro. In this study we demonstrated that protein expression of MKP5 was significantly downregulated in liver tissue of mice after I/R injury, and HepG2 cells subjected to H/R injury. MKP5 KO or knockdown significantly increased liver injury, as demonstrated by elevated serum transaminases, hepatocyte necrosis, infiltrating inflammatory cells, secretion of pro-inflammatory cytokines, apoptosis, oxidative stress*.* Conversely, MKP5 overexpression significantly attenuated liver and cell injury. Furthermore, we showed that MKP5 exerted its protective effect by inhibiting c-Jun N-terminal kinase (JNK)/p38 activity, and its action was dependent on Transforming growth factor-β-activated kinase 1 (TAK1) activity. According to our results, MKP5 inhibited the TAK1/JNK/p38 pathway to protect liver from I/R injury. Our study identifies a novel target for the diagnosis and treatment of liver I/R injury.

## Introduction

Liver ischemia/reperfusion (I/R) injury refers to the phenomenon that occurs after a period of ischemia in liver tissue during liver transplantation, hepatectomy, trauma, and hemorrhagic shock; the liver dysfunction is further aggravated after the recovery of blood supply^1,2^. At present, although liver surgical techniques are improving and surgical safety has been relatively enhanced, liver I/R injury is still an important concern that affects the incidence of perioperative complications and mortality of patients^3,4^. Currently, surgical intervention methods such as ischemic preconditioning and remote ischemic preconditioning, as well as drug intervention methods such as free radical scavengers, calcium channel blockers, hormones, or traditional Chinese medicine, are often used to alleviate liver I/R injury and improve patient survival rates ^5–9^. However, there are limited clinically recognized effective drugs for the treatment of liver I/R injury, and ischemic preconditioning is only effective in some young people^10–12^. Therefore, further understanding the pathogenesis of liver I/R injury and exploring new therapeutic strategies and targets for liver I/R injury is of great importance in clinical research.

The pathogenesis of liver I/R injury is characterized by many factors, including metabolic acidosis, calcium overload, mitochondrial damage, oxidative stress, apoptosis, activation of immune cells, among others^13–15^. Moreover, a number of signaling pathways are altered during liver I/R injury, among which the activation of mitogen-activated protein kinase (MAPK) signaling pathway contributes significantly to liver injury. Inhibiting MAPK pathway activation can significantly reduce liver I/R injury^16–20^. Therefore, finding new ways to regulate MAPK signaling has become an important consideration in treating liver I/R injury. MKP5 belongs to the MAPK phosphatase (MKP) family^21^. The MKP family inactivates target kinases through dephosphorylation of phosphoserine/threonine and phosphotyrosine residues, negatively regulating MAPK signaling, which is closely related to a variety of human diseases^21–23^. Previous studies have shown that in LPS-induced acute lung injury models, MAPK pathway activation is increased after MKP5 knockout, resulting in increased release of inflammatory factors and aggravated lung injury^24^. In addition, MKP5 may regulate the activity of JNK/p38, members of the MAPK signaling pathway, to reduce lipotoxicity and glucolipotoxicity-induced islet β-cell dysfunction and apoptosis^25,26^. Due to the important role of MAPK signaling pathway in liver I/R injury and the regulatory role of MKP5 on MAPK signaling pathway, we aimed to explore the potential effects of MKP5 in liver I/R injury.

## Materials and methods

### Animals

Male C57BL/6 J mice (6–8 weeks) were obtained from the Experimental Animal Center of Zhengzhou University. MKP5 knockout (MKP5 KO) mice with a C57BL/6 J background were provided by Prof. Yinming Liang from Xinxiang Medical College. All animals were housed in a specific pathogen-free environment with free access to food and water and a 12-h dark/light cycle. To overexpress MKP5 in liver, we transduced adeno-associated virus (AAV) 8 system carrying GFP scramble (AAV-GFP, as a negative control) or MKP5 (AAV-MKP5, designed and synthesized by GeneChem, Shanghai, China) into mice at a dose of 1 × 10^12^ viral genomes (200μL per mice) through tail-vein injection. I/R injury model was performed 4 weeks after virus injection. The study was carried out in compliance with the ARRIVE guidelines. Animal experiments were approved by the Ethics Committee of the First Affiliated Hospital of Zhengzhou University and cared for in accordance with the National Institutes of Health Guide for Laboratory Animals.

### Liver I/R model

We established a 70% I/R model in the mouse liver as previously described^27^. Briefly, after the mice were anesthetized with sodium pentobarbital, the abdominal cavity was opened, and the blood vessels of the left and middle hepatic lobes were separated and clamped with vascular clips. After 1.5 h of ischemia, the vascular clips were removed to start reperfusion. Mice in the sham-operated group received the same procedure without vessel clipping. Blood and liver tissue samples were collected after different time points of reperfusion.

### Liver damage assessment

After reperfusion, the blood samples of mice were collected, and the supernatant was collected after centrifugation at 3500 × g for 5 min, and the serum levels of alanine aminotransferase (ALT), aspartate aminotransferase (AST) and lactate dehydrogenase (LDH) (JianChen Bioengineering Institute, Nanjing, China) were measured according to the instructions of the detection kit.

### Hematoxylin and eosin staining

Liver tissues were fixed with 10% formalin, embedded in paraffin, and cut into 5-μm thick paraffin sections, and stained with hematoxylin and eosin according to the kit instructions (Servicebio, Wuhan, China). The stained sections were photographed and analyzed under an inverted optical microscope (Olympus, Tokyo, Japan).

### Terminal deoxynucleotidyl transferase dUTP nick-end labeling (TUNEL) assay

Paraffin-embedded liver tissues were cut into 5-μm thick paraffin sections, and apoptosis was detected according to the instructions of the TUNEL kit (Servicebio, Wuhan, China). After TUNEL staining, the slices were sealed with anti-fluorescent quenching agent, and then observed and photographed with a fluorescence microscope (Olympus, Tokyo, Japan). The number of TUNEL positive (the nuclei are stained red) cells were recorded from five non-overlapping fields in each slice, and the apoptosis rate was calculated.

### Immunohistochemical staining

Paraffin-embedded liver tissue sections were treated with xylene dewaxing and gradient ethanol dehydration, followed by repair solution, 3% H_2_O_2_ inactivation of endogenous peroxidase, and sections were sealed with 10% goat serum. Tissue sections were incubated with Ly6g (1:200, Servicebio, Wuhan, China) and F4/80 (1:200, Servicebio, Wuhan, China) primary antibodies overnight at 4 °C. After washing with PBS for three times, the sections were incubated with goat anti-rabit secondary antibody at room temperature for 30 min, followed by dropwise addition of DAB chromogenic solution. The sections were re-stained with hematoxylin, sealed with neutral balsam, and observed and photographed under the microscope.

### Quantitative real-time polymerase chain reaction (RT-PCR)

Total RNA was extracted with TriQuick reagent (Solarbio, Beijing, China), and cDNA was synthesized according to the instructions of reverse transcription kit (Vazyme, Nanjing, China). The reaction system was prepared for amplification according to the instructions of the RT-PCR kit (Vazyme, Nanjing, China). Amplification conditions were as follows: Pre-denaturation at 95˚C for 30 s, denaturation at 95˚C for 5 s, annealing and extension at 60˚C for 30 s, for a total of 40 cycles. The results were analyzed by the 2^−△△Ct^ method, and the expression of mRNA was expressed as a ratio to glyceraldehyde 3-phosphate dehydrogenase (GAPDH). Primer sequences are shown in Supplementary Table 1.

### Western blotting

The total protein in liver tissues and cells were extracted with RIPA reagent (Solarbio, Beijing, China), and the protein concentration was detected by BCA kit (Solarbio, Beijing, China), and then separated by 10% or 12% sodium dodecyl sulfonate-polyacrylamide gel electrophoresis. After electrophoresis, the gel was transferred to a PVDF membrane and blocked in 5% skimmed milk powder for 1 h at room temperature. Membranes were incubated with primary antibodies at 4 °C overnight. Membranes were washed with TBST and incubated with goat anti-rabbit or anti-mouse secondary antibody at 37 °C for 1 h. The ECL chemiluminescence reagent (NCM Biotech, Suzhou, China) was used for development, and the gel imaging system (Bio-Rad, CA, USA) was used for exposure and photography. The information for all antibodies used are described in Supplementary Table 2.

### Oxidative stress analysis

For DHE Staining, after reperfusion, liver tissue samples were taken and embedded with optimal cutting temperature compound, frozen and fixed quickly. Tissue specimens were cut into 10 μm sections, and then stained with 10 μM dihydroethidium (DHE) fluorescent probe and incubated at room temperature for 1 h in the dark. After staining nuclei with DAPI, the sections were observed and photographed with a fluorescence microscope. For MDA, SOD and GSH detection, after weighing the mouse liver tissue, add 0.9% normal saline pre-cooled at 4 °C according to the ratio of mass (g) : volume (mL) to 1:9 to prepare 10% liver homogenate, centrifuged at 8000 × g for 10 min to collect the supernatant and use the BCA protein concentration assay kit to quantify the protein concentration, and operate accordance with the kit instructions to detect the levels of MDA, SOD and GSH (Solarbio, Beijing, China) in the liver tissue of each group.

### Cell culture and cell H/R model

HepG2 cells were purchased from the cell bank of Chinese Academy of Sciences and cultured with Dulbecco’s modified Eagle medium (Solarbio, Beijing, China) supplemented with 10% fetal bovine serum (Gibco, CA, USA). Hypoxic reoxygenation (H/R) injury was induced by replacing the medium with glucose-free and serum-free medium (Procell, Wuhan, China) and incubating the cells in a three-gas incubator (94%N_2_, 5%CO_2_, 1%O_2_)^28^. After 6 h of hypoxia, the cell medium was replaced with fresh complete medium and placed in a normoxic incubator (95% air + 5%CO_2_ mixture) for reoxygenated at different time points. To explore the role of TAK1 and MKP5 on liver cell H/R injury, TAK1 inhibitor 5Z-7-ox (1 μM) (MedChemExpress LLC, Shanghai, China) was administrated to cells 1 h prior to H/R. For a negative control, cells were treated with dimethyl sulfoxide (DMSO; Solarbio, Beijing, China) vehicle only and then exposed to hypoxic conditions.

### Plasmid construction and cell transfection

The MKP5 overexpression (flag and flag-MKP5) and knockdown lentivirus plasmids (Negative control and sh-MKP5) were purchased from PPL, Inc.(Nanjing, China). MKP5 knockout lentivirus plasmid sequence are as follows, sh-1: CAATGAACCAAGCCGAGTGAT, sh-2: GACCATGACTGATGCTTATAA, sh-3: CAGGCTCTCTAAGTCCATCAA, Negative control: GTTCTCCGAACGTGTCACGTT. The lentiviral plasmid and packaging plasmid (CMV and VSVG) were transfected into HEK 293 T cells with Lipo3000 transfection reagent according to the instructions. The medium containing lentiviral particles was collected 48 h after transfection. The harvested viral particles were transfected into HepG2 cells. 24 h after viral transfection, cells with stable expression were obtained by screening with medium containing 2 μg/ml puromycin (Gibco, CA, USA) for 7 days.

### Cell injury assay

Cell counting kit-8 (CCK-8) assay and lactic dehydrogenase (LDH) content were used to assess cell injury. For CCK-8 assay, HepG2 cells were inoculated into 96-well plates at a cell density of 5 × 10^3^ cells/well, and H/R was performed when the cell density reached 80% confluency. After reoxygenation, 10 μL of CCK-8 reagent (Boster, Wuhan, China) was added to each well according to the instructions of the CCK-8 kit, and the absorbance of each well was detected at 450 nm with microplate reader after incubation for 1 h at 37 °C. The cell activity was calculated according to the absorbance value. For LDH assay, after reoxygenation, cell culture medium was collected and operated according to the instructions of the detection kit.

### Flow cytometry

After reoxygenation, cells were collected with trypsin without EDTA (Beyotime, Shanghai, China). After washing twice with ice-cold PBS, cells were resuspended in 200 μL of binding buffer, and 5 μL of AnnexinV-FITC and 5 μL of propidium iodide (Beyotime, Shanghai, China) were added, and the cells were incubated at room temperature for 15 min in the dark. An additional 400 μL of binding buffer was added and the cells were analyzed by flow cytometry.

### Statistical analysis

All data were analyzed using SPSS software (version 17.0, SPSS Inc., Chicago, IL, USA) and expressed as mean ± standard deviation. T-test was used for comparison between two groups, and one-way ANOVA analysis was used for comparison between more than two groups. *P* < 0.05 was considered statistically significant.

## Result

### MKP5 is involved in the regulation of liver I/R injury in mice

**Figure 1:**
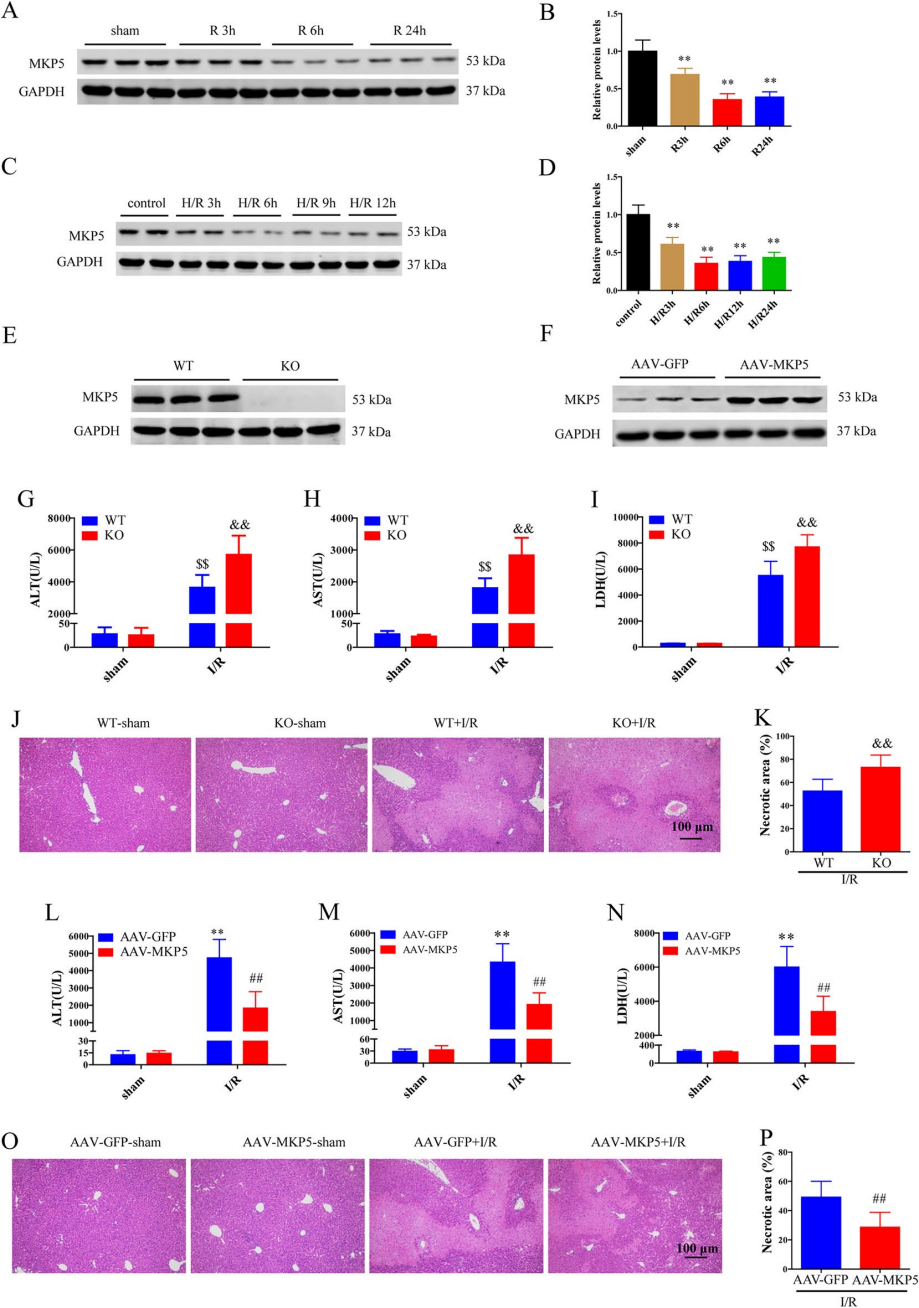
MKP5 is involved in the regulation of liver I/R injury in mice. (**A**–**B**) Protein expression levels and statistical analysis of MKP5 in mouse liver tissues subjected to 1.5 h ischemia and indicated time of reperfusion. (**C**–**D**) Protein expression levels and statistical analysis of MKP5 in HepG2 cells subjected to 6 h hypoxia and indicated time of reoxygenation. (**E**) MKP5 protein levels in livers from wild type (WT) and MKP5-knockout (KO) mice. (**F**) MKP5 protein levels in livers from AAV-GFP and AAV-MKP5 mice. (**G**–**I**) Serum ALT, AST and LDH levels in WT and MKP5-KO mice at 6 h after reperfusion. (**J**) Representative H&E-stained liver sections and (K) necrotic area statistics in WT and MKP5-KO mice at 6 h after reperfusion (scale bar, 100 μm). (**L**–**M**) Serum ALT, AST and LDH levels in AAV-GFP and AAV-MKP5 mice at 6 h after reperfusion. (**O**) Representative H&E-stained liver sections and (**P**) necrotic area statistics in AAV-GFP and AAV-MKP5 mice at 6 h after reperfusion (scale bar, 100 μm). **P<0.01 in sham group or normal group in figure B and D. *P < 0.05 and **P < 0.01 vs. AAV-GFP sham group; ^#^*P* < 0.05 and ^##^*P* < 0.01 vs. AAV-GFP I/R group; ^$^*P* < 0.05 and ^$$^*P* < 0.01 vs. WT sham group;^&^*P* < 0.05 and ^&&^*P* < 0.01 vs. WT I/R group in figures G–P.

To analyze the role of MKP5 in liver I/R injury, we evaluated the protein expression of MKP5 in liver tissues after I/R injury and in HepG2 cells challenged by H/R injury. We found that the protein expression of MKP5 was significantly downregulated in vivo and in vitro (Fig. 1A–D). The expression of MKP5 was the lowest at 6 h of reperfusion and 6 h of reoxygenation, and these two time points were selected for subsequent experiments. Next, we used MKP5 KO mice and AAV-MKP5 mice to investigate whether MKP5 regulates liver IR injury. Western blot analysis confirmed MKP5 KO or overexpression in the liver (Fig. 1E and F). Notably, compared to wildtype (WT) mice, liver IR-injured mice with MKP5 KO displayed significantly higher serum levels of ALT, AST and LDH (Fig. 1G–I). Additionally, liver sections from MKP5 KO mice showed more severe necrosis after liver I/R injury compared to WT mice (Fig. 1J and K). Furthermore, compared to AAV-GFP group mice, serum levels of AST, ALT and LDH (Fig. 1L–N), and liver tissue necrosis were reduced in AAV-MKP5 group mice (Fig. 1O and P). These results suggest that MKP5 protects the liver from injury caused by I/R.

### MKP5 reduces inflammatory response after liver I/R injury

**Figure 2:**
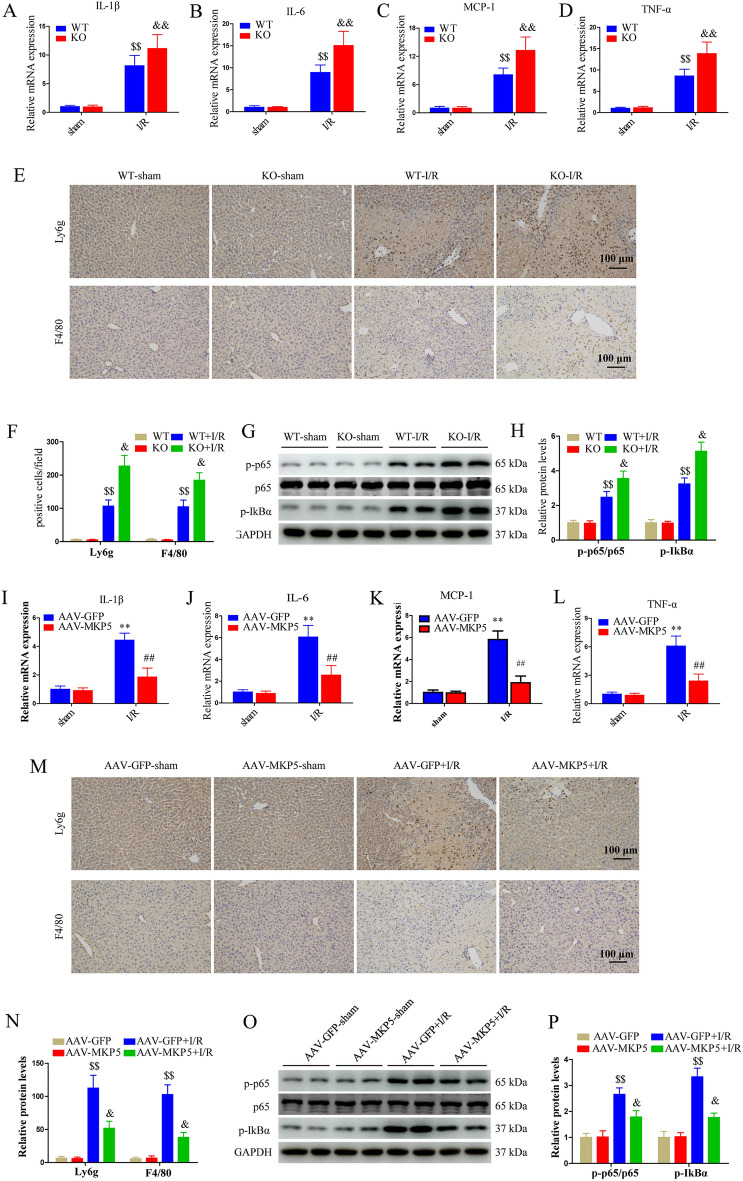
MKP5 reduces inflammatory response after liver I/R injury. (**A**–**D**) The mRNA expression of IL-1β, IL-6, TNF-α, and MCP-1 of liver tissues in WT and MKP5-KO mice at 6 h after reperfusion. (**E**) Representative Ly6g and F4/80 immunohistochemical staining of liver tissues and (**F**) statistical analysis in WT and MKP5-KO mice at 6 h after reperfusion (scale bar, 100 μm). (**G**) The protein expression of p-p65, p65 and p-IkBα and (**H**) protein quantitative analysis in WT and MKP5-KO mice. (**I**–**L**) The mRNA expression of IL-1β, IL-6, TNF-α, and MCP-1 of liver tissues in AAV-GFP and AAV-MKP5 mice at 6 h after reperfusion. (**M**) Representative Ly6g and F4/80 immunohistochemical staining of liver tissues and (**N**) statistical analysis in AAV-GFP and AAV-MKP5 mice at 6 h after reperfusion (scale bar, 100 μm). (**O**) The protein expression of p-p65, p65 and p-IkBα and (**P**) protein quantitative analysis in AAV-GFP and AAV-MKP5 mice. *P < 0.05 and **P < 0.01 vs. AAV-GFP sham group; ^#^*P* < 0.05 and ^##^*P* < 0.01 vs. AAV-GFP I/R group; ^$^*P* < 0.05 and ^$$^*P* < 0.01 vs. WT sham group; ^&^*P* < 0.05 and ^&&^*P* < 0.01 vs. WT I/R group.

During liver I/R injury, the inflammatory response plays a pivotal role in the overall I/R process, and inhibition of inflammation can effectively alleviate liver I/R injury. Therefore, we tested the role of MKP5 in the inflammatory response in our models. Compared to WT mice, mRNA expression of interleukin 6 (Il-6), interleukin 1β (IL-1β), tumor necrosis factor α (TNF-α), and monocyte chemoattractant protein-1 (MCP-1) were increased in liver tissues of MKP5 KO mice after I/R injury (Fig. 2A–D). In addition, after liver I/R injury, immunohistochemistry staining showed a significant increase in the number of infiltrating inflammatory cells (Ly6g^+^ cells and F4/80^+^) in MKP5 KO mice compared to WT mice (Fig. 2E and F). We further demonstrated that MKP5 KO enhanced the activation of the nuclear factor kappa B (NF-κB) signaling pathway, shown by elevated levels of nuclear factor of kappa light polypeptide gene enhancer in B-cells inhibitor alpha (IκBα) and p65 phosphorylation, compared with WT mice (Fig. 2G and H). In contrast, AAV-MKP5 group mice inhibited the expression of inflammatory factors, inflammatory cell infiltration, and NF-κB signaling pathway compared with AAV-GFP group mice (Fig. 2I–P). Collectively, these suggest that MKP5 is an important regulator of the inflammatory response during liver I/R injury.

### MKP5 attenuates apoptosis after liver I/R injury

**Figure 3:**
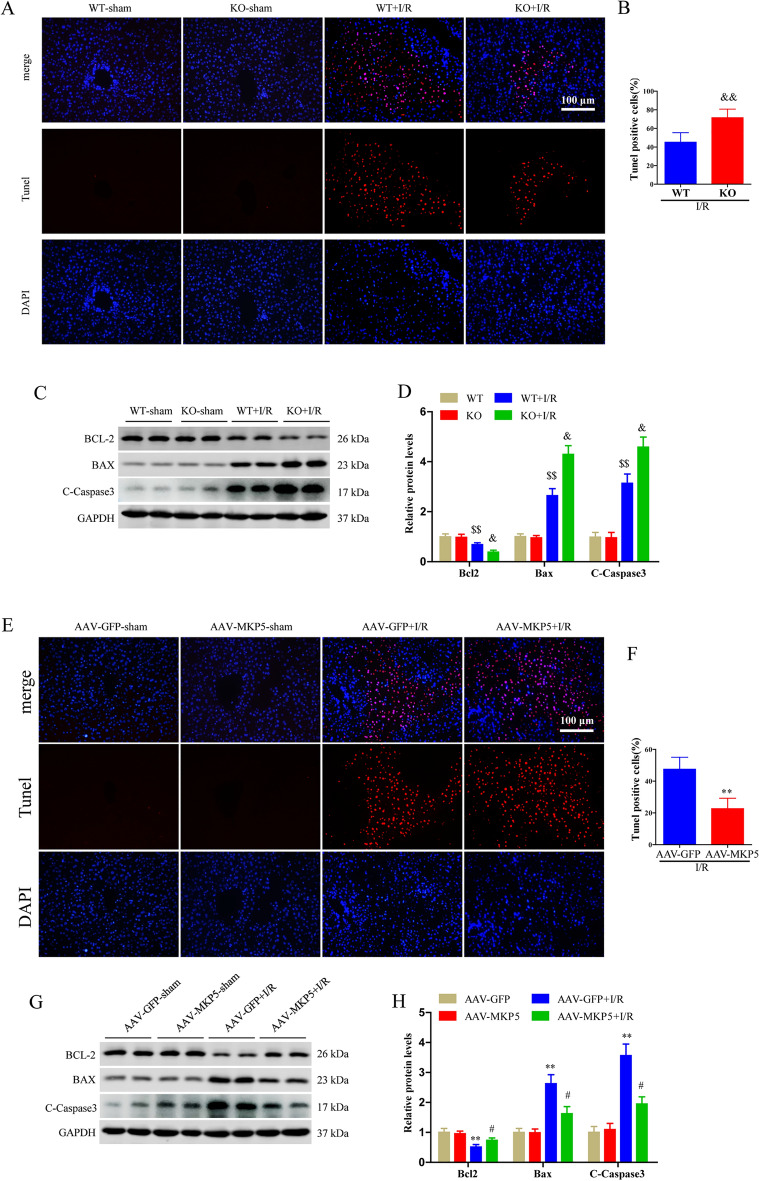
MKP5 attenuates apoptosis after liver I/R injury. (**A**–**B**) TUNEL staining and statistical analysis (scale bar, 100 μm) of liver tissues in WT and MKP5-KO mice (The nuclei of TUNEL positive cells are stained red.). (**C**) The protein expression of BCL-2, BAX and C-Caspase 3 and (**D**) protein quantitative analysis in WT and MKP5-KO mice. (**E**–**F**) TUNEL staining and statistical analysis (scale bar, 100 μm) of liver tissues in AAV-GFP and AAV-MKP5 mice. (**G**) The protein expression of BCL-2, BAX and C-Caspase 3 and (**H**) protein quantitative analysis in AAV-GFP and AAV-MKP5 mice.*P < 0.05 and **P < 0.01 vs. AAV-GFP sham group; ^#^*P* < 0.05 and ^##^*P* < 0.01 vs. AAV-GFP I/R group; ^$^*P* < 0.05 and ^$$^*P* < 0.01 vs. WT sham group; ^&^*P* < 0.05 and ^&&^*P* < 0.01 vs. WT I/R group.

Apoptosis during liver I/R injury is directly involved in liver injury. TUNEL staining of liver tissue after I/R injury showed a significant increase in the number of apoptotic cells in MKP5 KO mice compared with WT mice (Fig. 3A and B). In addition, the expression of pro-apoptotic protein cleaved caspase-3 and B-cell lymphoma protein 2 (BCL-2)-associated X protein (BAX) were also significantly increased, while the expression of anti-apoptotic protein BCL-2 was significantly decreased in MKP5 KO mice after I/R injury (Fig. 3C and D). In contrast, compared to AAV-GFP group mice, AAV-MKP5 group mice had decreased apoptosis after liver I/R injury, demonstrated by decreased TUNEL-positive cell numbers, reduced protein expression of BAX and cleaved caspase-3, and increased protein expression of BCL-2 (Fig. 3E–H). Overall, these datas suggest that MKP5 inhibits hepatocyte apoptosis in liver I/R injury.

### MKP5 reduces oxidative stress after liver I/R injury

**Figure 4:**
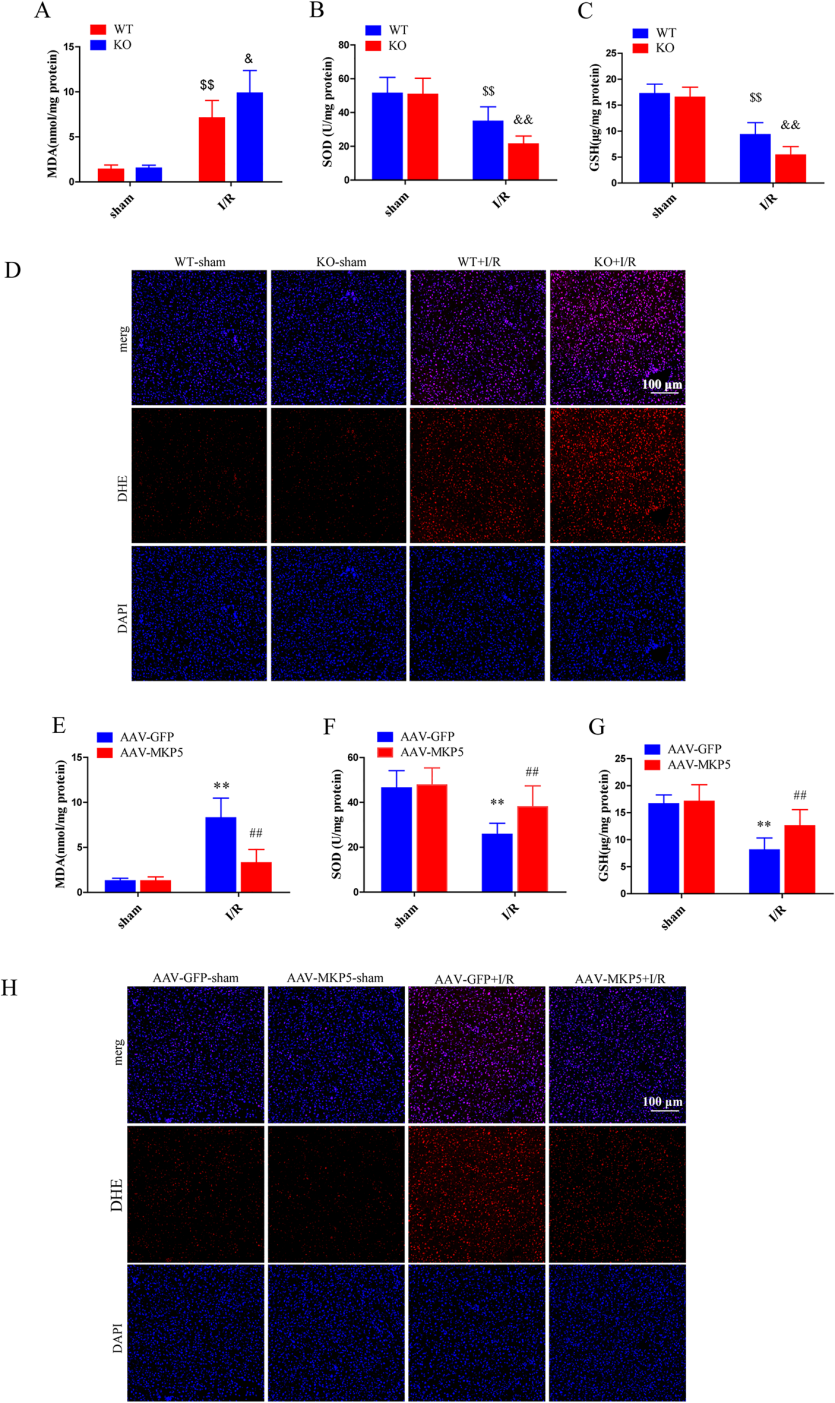
MKP5 reduces oxidation stress after liver I/R injury. (**A**–**D**) Liver MDA content, SOD activity, GSH activity, representative DHE stained liver sections in WT and MKP5-KO mice at 6 h after reperfusion. (**E**–**H**) Liver MDA content, SOD activity, GSH activity, representative DHE stained liver sections in AAV-GFP and AAV-MKP5 mice at 6 h after reperfusion. *P < 0.05 and **P < 0.01 vs. AAV-GFP sham group; ^#^*P* < 0.05 and ^##^*P* < 0.01 vs. AAV-GFP I/R group; ^$^*P* < 0.05 and ^$$^*P* < 0.01 vs. WT sham group; ^&^*P* < 0.05 and ^&&^*P* < 0.01 vs. WT I/R group.

SOD, GSH and MDA are important indexes of oxidative stress, DHE staining can be used to detect the content of ROS. Compared with the sham group, the activities of SOD and GSH in liver tissue were significantly decreased, and the content of MDA and ROS production were significantly increased after liver I/R (Fig. 4A–C). Compared with WT mice, MKP5 KO mice had significantly increased MDA content, ROS production, and decreased SOD and GSH activity in the liver after I/R injury (Fig. 4A–D). In contrast, MKP5 overexpression reduced significantly the MDA content, ROS production and increased SOD and GSH activity compared to AAV-GFP group mice (Fig. 4E and H). These results demonstrate that MKP5 suppresses oxidative stress after liver I/R.

### MKP5 attenuates cell damage after H/R injury

**Figure 5:**
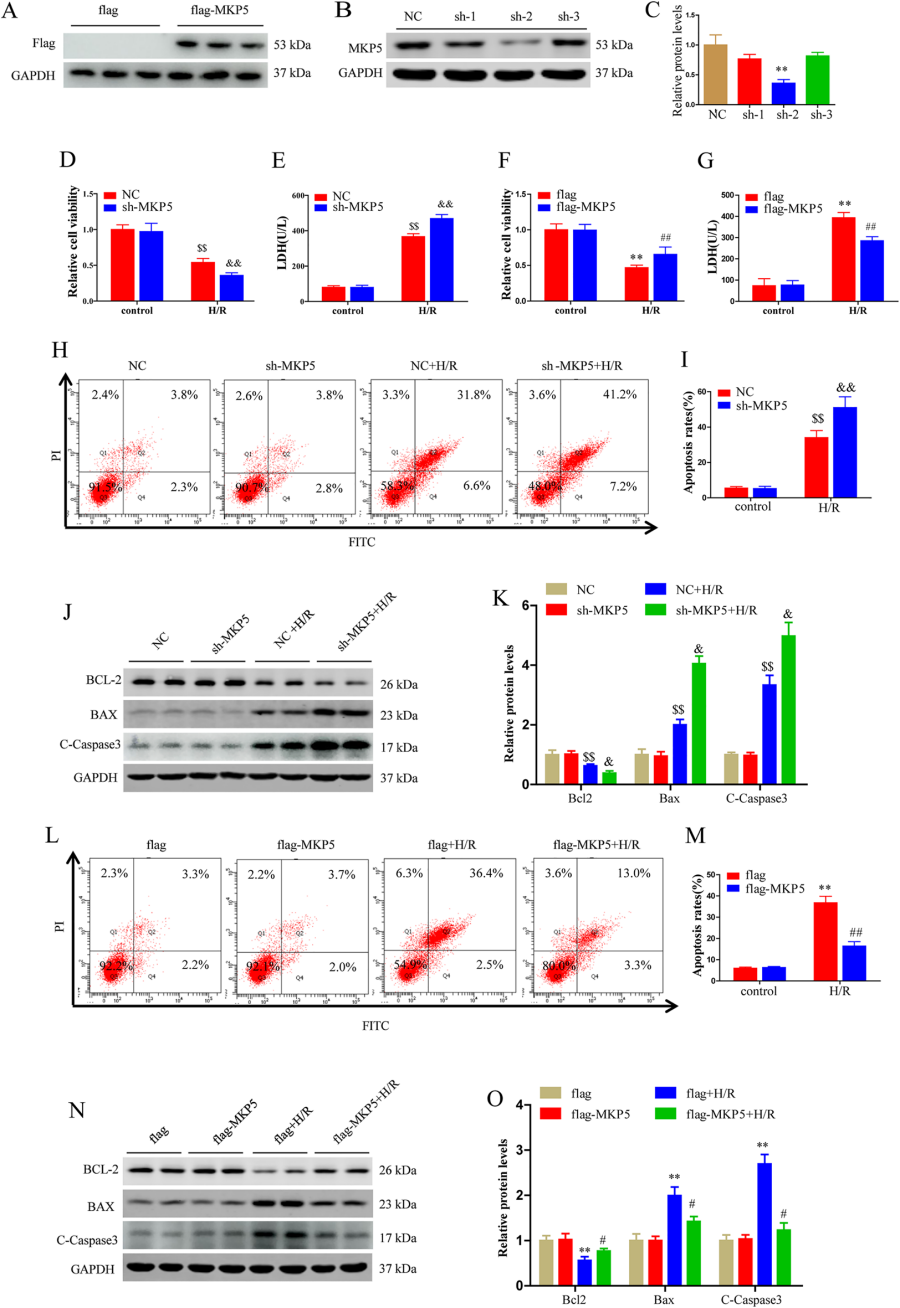
MKP5 attenuates cell damage after H/R injury. (**A**) Detection of protein expression after MKP5 overexpression. (**B**–**C**) Protein expression levels and statistical analysis of MKP5 after transfection with knockdown lentivirus. (**D**–**E**) Cell viability and LDH content in NC and sh-MKP5 HepG2 cells at 6 h after reoxygenation. (**F**–**G**) Cell viability and LDH content in flag and flag-MKP5 HepG2 cells at 6 h after reoxygenation. (**H**–**I**) Cell apoptosis in NC and sh-MKP5 HepG2 cells at 6 h after reoxygenation was determined by flow cytometry. (**J**) The protein expression of BCL-2, BAX and C-Caspase 3 and (**K**) statistical analysis in NC and sh-MKP5 HepG2 cells at 6 h after reoxygenation. (**L**–**M**) Cell apoptosis in flag and flag-MKP5 HepG2 cells at 6 h after reoxygenation was determined by flow cytometry. (**N**) The protein expression of BCL-2, BAX and C-Caspase 3 and (**O**) statistical analysis in flag and flag-MKP5 HepG2 cells at 6 h after reoxygenation.^**^P<0.05 vs. NC group in figure C. ^$^*P* < 0.05 and ^$$^*P* < 0.01 vs. NC control group; ^&^*P* < 0.05 and ^&&^*P* < 0.01 vs. NC H/R group; ^*^P < 0.05 and ^**^P < 0.01 vs. flag control group; ^#^*P* < 0.05 and ^##^*P* < 0.01 vs. flag H/R group in figures D-O.

We further examined the function of MKP5 in HepG2 cells subjected to H/R stimulation. Lentivirus containing either MKP5 shRNA or MKP5 expression plasmid were used to establish MKP5 knockdown or overexpression cells, respectively. Cell viability, LDH content in medium, cell apoptosis, and the expression of apoptosis-related proteins were detected. The knockdown and overexpression of MKP5 was verified by western blot (Fig. 5A–C), the lentivirus sh-2 sequence knockdown effect was the most obvious, which was used for subsequent experiments. Compared to NC group cells, MKP5 knockdown cells exposed to H/R resulted in significantly reduced cell viability, significantly increased LDH content and apoptosis (Fig. 5D–E and H–K). However, compared with flag group cells, H/R-treated HepG2 cells overexpressing MKP5 showed increased viability, LDH production, and decreased apoptosis (Fig. 5F–G and L–O). These results suggest that MKP5 has a protective effect against H/R-induced hepatocyte injury.

### MKP5 inhibits the TAK1/JNK/p38 signaling pathways after liver I/R injury

**Figure 6:**
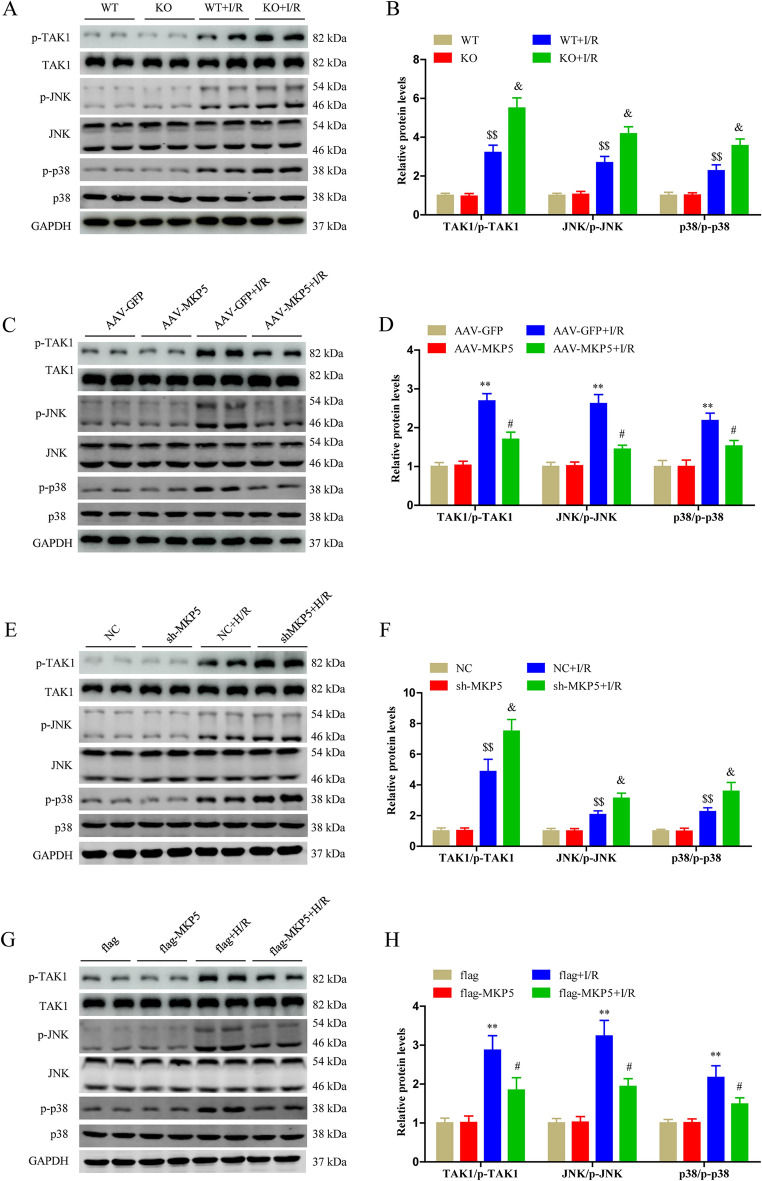
MKP5 inhibits the TAK1/JNK/p38 signaling pathways after liver I/R injury. (**A**–**B**) Protein expression levels and statistical analysis of P-TAK1, TAK1, p-JNK, JNK, p-p38 and p-38 in in WT and MKP5-KO mice at 6 h after reperfusion. (**C**–**D**) Protein expression levels and statistical analysis of p-TAK1, TAK1, p-JNK, JNK, p-p38 and p-38 in AAV-GFP and AAV-MKP5 mice at 6 h after reperfusion. (**E**–**F**) Protein expression levels and statistical analysis of p-TAK1, TAK1, p-JNK, JNK, p-p38 and p-38 in NC and sh-MKP5 HepG2 cells at 6 h after reoxygenation. (**G**–**H**) Protein expression levels and statistical analysis of p-TAK1, TAK1, p-JNK, JNK, p-p38 and p-38 in in flag and flag-MKP5 HepG2 cells at 6 h after reoxygenation. For *P < 0.05 and **P < 0.01 vs. AAV-GFP sham or flag control group; ^#^*P* < 0.05 and ^##^*P* < 0.01 vs. AAV-GFP I/R or flag + H/R group; ^$^*P* < 0.05 and ^$$^*P* < 0.01 vs. WT sham or NC control group; ^&^*P* < 0.05 and ^&&^*P* < 0.01 vs. WT I/R or NC + H/R group.

The MAPK signaling pathway is involved in regulating inflammation, apoptosis and oxidative stress after liver I/R injury. MKP5, a member of the MKP family, negatively regulates the activity of the MAPK pathway. Therefore, we speculated that MKP5 might protect liver from I/R injury by inhibiting the MAPK pathway. As shown in Fig. 6A and B, compared to WT mice, the protein levels of phosphorylated p38 and JNK were both upregulated in MKP5 KO mouse liver tissues after I/R injury. We then examined the expression of TAK1 an upstream protein of the MAPK signaling pathway. MKP5 KO mice had increased phosphorylated TAK1 expression in liver tissues after I/R injury compared to WT mice (Fig. 6A and B). In contrast, compared to AAV-GFP group mice, AAV-MKP5 group mice had reduced phosphorylation of TAK1, and reduced expression of p38 and JNK (Fig. 6C and D). Consistent with the in vivo results, we found the similar trend in HepG2 cells subjected to H/R injury (Fig. 6E–H). Together, these observations suggeste that in hepatocytes, MKP5 inhibits the activation of TAK1/JNK/p38 pathway during liver I/R-induced liver injury.

### Effect of MKP5 on liver I/R depends on TAK1 activity

**Figure 7:**
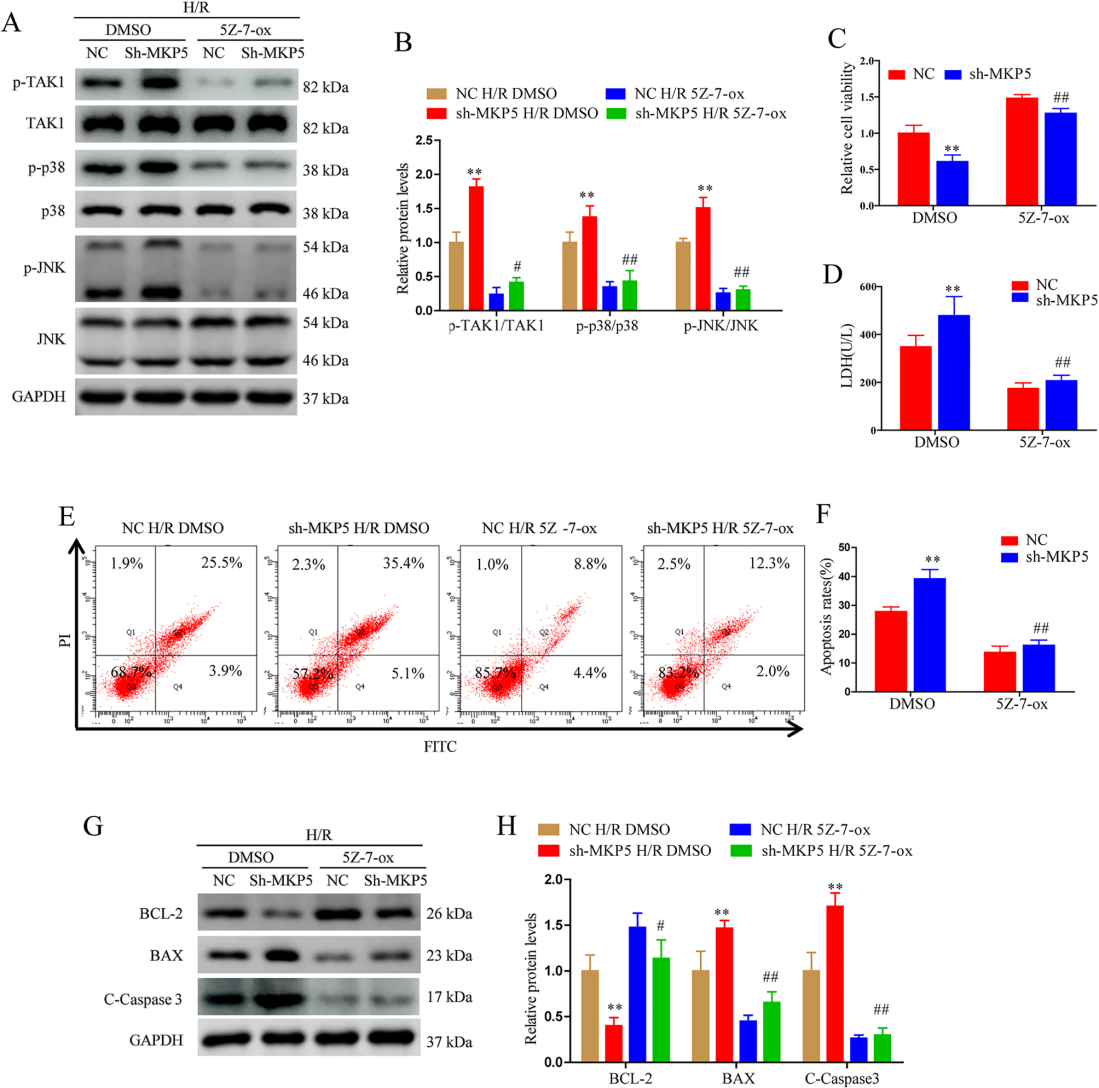
The effect of MKP5 on liver I/R depends on TAK1 activity. (**A**–**B**) Protein expression levels and statistical analysis of P-TAK1, TAK1, p-JNK, JNK, p-p38 and p-38 in NC and sh-MKP5 HepG2 cells pretreated with DMSO or 5Z-7-ox at 6 h after reoxygenation. (**C**–**D**) Cell viability and LDH content in NC and sh-MKP5 HepG2 cells pretreated with DMSO or 5Z-7-ox at 6 h after reoxygenation. (**E**–**F**) Cell apoptosis in NC and sh-MKP5 HepG2 cells pretreated with DMSO or 5Z-7-ox at 6 h after reoxygenation. (**G**) Apoptosis-related proteins and (**H**) statistical analysis in NC and sh-MKP5 HepG2 cells pretreated with DMSO or 5Z-7-ox at 6 h after reoxygenation. *P < 0.05 and **P < 0.01 vs. NC HR DMSO group; ^#^*P* < 0.05 and ^##^*P* < 0.01 vs. sh-MKP5 H/R DMSO group.

To evaluated whether the protective effect of MKP5 was dependent on TAK1, we used a specific TAK1 inhibitor (5Z-7-ox) to block the activity of TAK1 and inhibit activation of downstream JNK/p38 signalling in MKP5 knockdown HepG2 cells prior to H/R injury. Compared with vehicle treated cells, 5Z-7-ox inhibited the phosphorylation of TAK1 and JNK/p38 in MKP5 knockdown HepG2 cells subjected to H/R challenge (Fig. 7A and B). Furthermore, TAK1 inhibition also reversed the cell viability decrease, LDH release and apoptosis caused by MKP5 knockdown in HepG2 cells subjected to H/R (Fig. 7C–H). These observations suggest that TAK1 inhibition could eradicate the effects of MKP5 knockdown on H/R- induced cell injury, suggesting that TAK1 mediates the protective effect of MKP5 on liver I/R injury.

## Discussion

In the present study, we found that MKP5 expression was significantly reduced after liver I/R injury and in HepG2 cells H/R. MKP5 knockdown exacerbated liver injury via promoting inflammation, apoptosis, and oxidative stress. In contrast, overexpression of MKP5 significantly attenuated liver injury. Further analysis showed that MKP5 protected against liver I/R injury through regulating the TAK1/JNK/p38 pathway, and inhibition of TAK1 activity could reduce the cellular injury caused by MKP5 knockdown in HepG2 cells subjected to H/R.

Inflammation and apoptosis play an important roles in liver I/R injury^16,17,29^. During liver I/R injury, activated Kupffer cells produce reactive oxygen species, pro-inflammatory cytokines, chemokines, and adhesion molecules, leading to cell apoptosis and tissue damage^8^. Consequently, the damaged hepatocytes promote more inflammatory cell infiltration, leading to a continuous vicious cycle that aggravates liver injury^13,15^. Targeting inflammatory response and apoptosis pathways during this process will significantly improve the prognosis of clinical I/R injury^29^. In our study, MKP5 KO led to a significant increase in the expression of pro-inflammatory factors (IL-1β, IL-6, TNF-α and MCP-1), and Ly6g and F4/80 positive cell infiltration. Furthermore, NF-κB pathway activation was more pronounced in liver tissues of MKP5 KO mice compared to the WT mice. TUNEL staining and apoptosis-related proteins BAX, BCL-2, and cleaved caspase-3 showed that MKP5 KO increased liver I/R-induced apoptosis. In contrast, compared with AAV-GFP group mice exposed to I/R, AAV-MKP5 group mice had reduced liver inflammation and apoptosis. Thus, we concluded that MKP5 could significantly reduce liver I/R-induced inflammation and apoptosis.

A large number of oxygen radicals are generated in liver tissue during I/R injury^30^. Oxygen radicals and unsaturated fatty acids on biological membranes undergo lipid peroxidation to form lipid peroxides such as MDA, which is cytotoxic and can aggravate cell membrane damage and affect normal physiological functions of cells^30^. The important antioxidant enzymes SOD and GSH can scavenge oxygen free radicals and reduce the damage of lipid peroxidation in cells. Therefore, MDA content, SOD and GSH activity are often used to reflect the oxidative stress level of the body or tissues^31,32^. Dihydroethidium (DHE) can freely enter the cell and is oxidized by intracellular ROS to produce red fluorescence, which can be used to judge the amount and change of cellular ROS content^33,34^. Qian et al. showed that MKP5 attenuates LPS-induced vascular injury by inhibiting ROS production^35^. Zhao et al. demonstrated that MKP5 alleviates PA-induced islet β cell dysfunction by inhibiting oxidative stress^26^. These findings are consistent with the results shown in our study that MKP5 KO resulted in increased ROS production and MDA content, and decreasd SOD and GSH activity in liver tissues after I/R injury. While overexpression of MKP5 decreased the ROS production and MDA content, and increased SOD and GSH activity, suggesting that MKP5 inhibits oxidative stress after liver I/R injury.

The MAPK family consists of extracellular regulatory protein kinases (ERK), JNK, and p38, which are involved in the physiological processes of cell proliferation, apoptosis, inflammatory response, oxidative stress and other physiological processes in liver I/R injury^36,37^. Inhibition of MAPK activity is a potential strategy for alleviating liver I/R injury^16–20,29,37^. In the present study, we found that JNK/p38 phosphorylation was significantly increased in MKP5 KO mice and MKP5 knockdown HepG2 cells, whereas JNK/p38 phosphorylation was decreased in the MKP5 overexpression models. We further examined the changes in the expression of TAK1, a protein upstream of JNK/p38, and MKP5 KO or knockdown significantly increased TAK1 phosphorylation, whereas MKP5 overexpression inhibited the TAK1 phosphorylation. Inhibition of TAK1 activity with a TAK1 inhibitor reversed cell damage caused by MKP5 knockdown. Together, these observations indicate that MKP5 inhibits TAK1 phosphorylation and downstream JNK and p38 activation to alleviate liver I/R injury.

However, this study also has some limitations. In this study, MKP5 gene knockout mice and MKP5 mice in adeno-associated virus overexpressed liver were used. If hepatocyte-specific knockout and overexpression mice were used, the experimental results would be more convincing. In addition, whether MKP5 regulates TAK1 directly or through other means needs to be further investigated.

To conclude, we found that MKP5 ameliorates liver I/R injury through the regulation of inflammation, apoptosis, and oxidative stress. The protective effect of MKP5 is dependent on inhibition of the TAK1/JNK/p38 signaling pathways. Therefore, targeting MKP5 may offer a promising therapeutic approach for liver I/R injury.

## Supplementary Information

Supplementary Table 1.

Supplementary Table 2.

Supplementary Information.

## Data Availability

The data that support the findings of this study are available from the corresponding author on reasonable request.
