# Supplementary material for: Mitogen activated protein kinase phosphatase 5 alleviates liver ischemia–reperfusion injury by inhibiting TAK1/JNK/p38 pathway
Source: Sci Rep. 2023 Jul 10;13:11110. doi: 10.1038/s41598-023-37768-9 (PMC10333288; doi:10.1038/s41598-023-37768-9)
Supplement: Supplementary file 1 — Supplementary Table 1. [file 41598_2023_37768_MOESM1_ESM.docx]

Table 1. Gene-specific quantitative-polymerase chain reaction primers

| Primer | Primer sequence |
| --- | --- |
| IL-1β F | 5ʹ-GCTTCAGGCAGGCAGTATCA-3ʹ |
| IL-1β R | 5ʹ-AGTCACAGAGGATGGGCTCT-3ʹ |
| IL-6 F | 5ʹ-AGAGACTTCCATCCAGTTGCC-3ʹ |
| IL-6 R | 5ʹ-TCCTCTGTGAAGTCTCCTCTCC-3ʹ |
| TNF-α F | 5ʹ-AGCCGATGGGTTGTACCTTG-3ʹ |
| TNF-α R | 5ʹ-ATAGCAAATCGGCTGACGGT-3ʹ |
| MCP-1 F | 5ʹ-ATCTGCCCTAAGGTCTTCAGC-3ʹ |
| MCP-1 R | 5ʹ-AGGCATCACAGTCCGAGTCA-3ʹ |
| GAPDH F | 5ʹ-CTGCCCAGAACATCATCCCT-3ʹ |
| GAPDH R | 5ʹ-TACTTGGCAGGTTTCTCCAGG-3ʹ |
