# Supplementary material for: Mitogen activated protein kinase phosphatase 5 alleviates liver ischemia–reperfusion injury by inhibiting TAK1/JNK/p38 pathway
Source: Sci Rep. 2023 Jul 10;13:11110. doi: 10.1038/s41598-023-37768-9 (PMC10333288; doi:10.1038/s41598-023-37768-9)
Supplement: Supplementary file 2 — Supplementary Table 2. [file 41598_2023_37768_MOESM2_ESM.docx]

| Antibody | company | Catalog number | source | Concentration |
| --- | --- | --- | --- | --- |
| MKP5  p65  p-p65  p- IκBα  BAX | Santacruz  Proteintech  CST  CST  Proteintech | sc-374276  10745-1-AP  3033  2859  50599-2-Ig | Mouse  Rabbit  Rabbit  Rabit  Rabbit | 1:500  1:1000  1:1000  1:1000  1:1000 |
| BCL2 | HUABIO | ET1702-53 | Rabbit | 1:1000 |
| Cleaved caspase3 | CST | 9664 | Rabbit | 1:1000 |
| p-JNK  JNK | CST  Proteintech | 4668  24164-1-AP | Rabbit  Rabbit | 1:1000  1:1000 |
| p-p38 | CST | 4511 | Rabbit | 1:1000 |
| p38  p-TAK1  TAK1  GAPDH | HUABIO  ABclonal  HUABIO  Proteintech | ET1702-65  AP1222  ET1705-14  60004-1-Ig | Rabbit  Rabbit  Rabbit  Mouse | 1:10000  1:1000  1:1000  1:5000 |

Table 2 primary antibody information
