# Supplementary material for: Mitogen activated protein kinase phosphatase 5 alleviates liver ischemia–reperfusion injury by inhibiting TAK1/JNK/p38 pathway
Source: Sci Rep. 2023 Jul 10;13:11110. doi: 10.1038/s41598-023-37768-9 (PMC10333288; doi:10.1038/s41598-023-37768-9)
Supplement: Supplementary file 3 — Supplementary Information. [file 41598_2023_37768_MOESM3_ESM.pdf]

Figure 1A

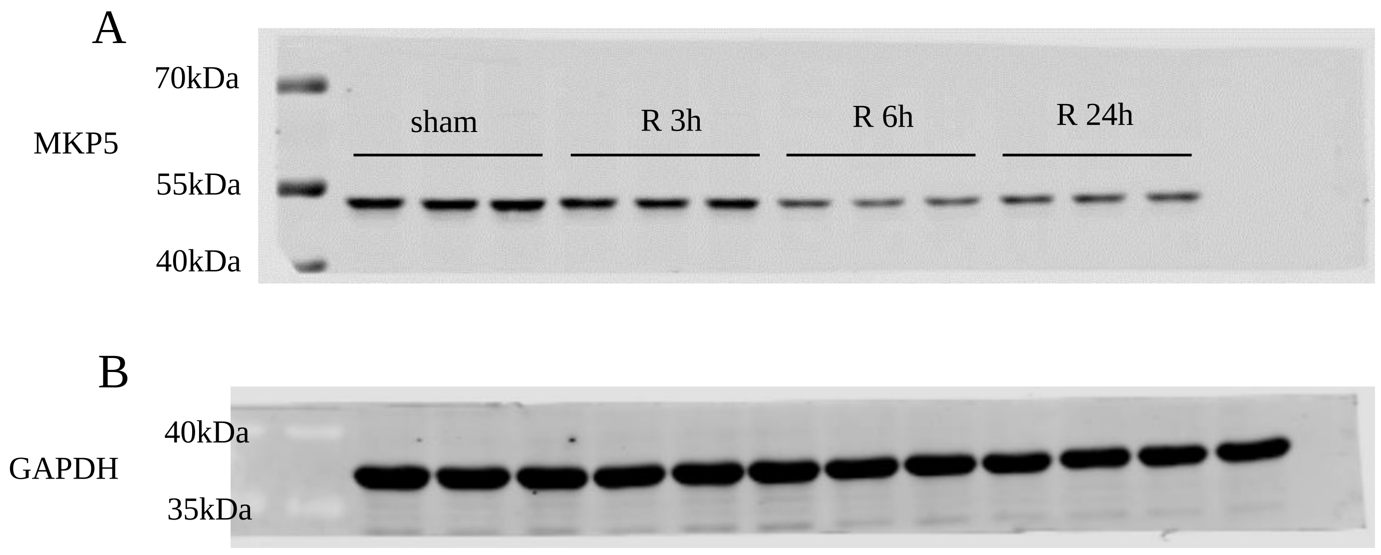


(A) MKP5 protein bands in Figure 1A; (B) GAPDH protein bands in Figure 1A.

Figure 1C

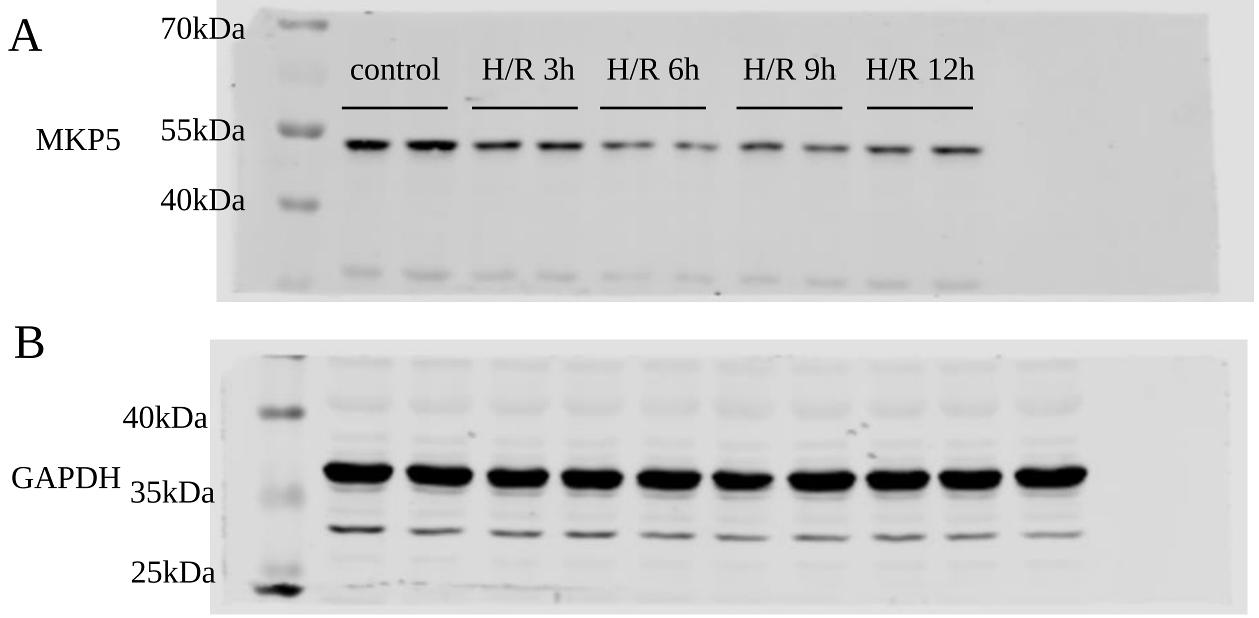


(A) MKP5 protein bands in Figure 1C; (B) GAPDH protein bands in Figure 1C.

Figure 1E

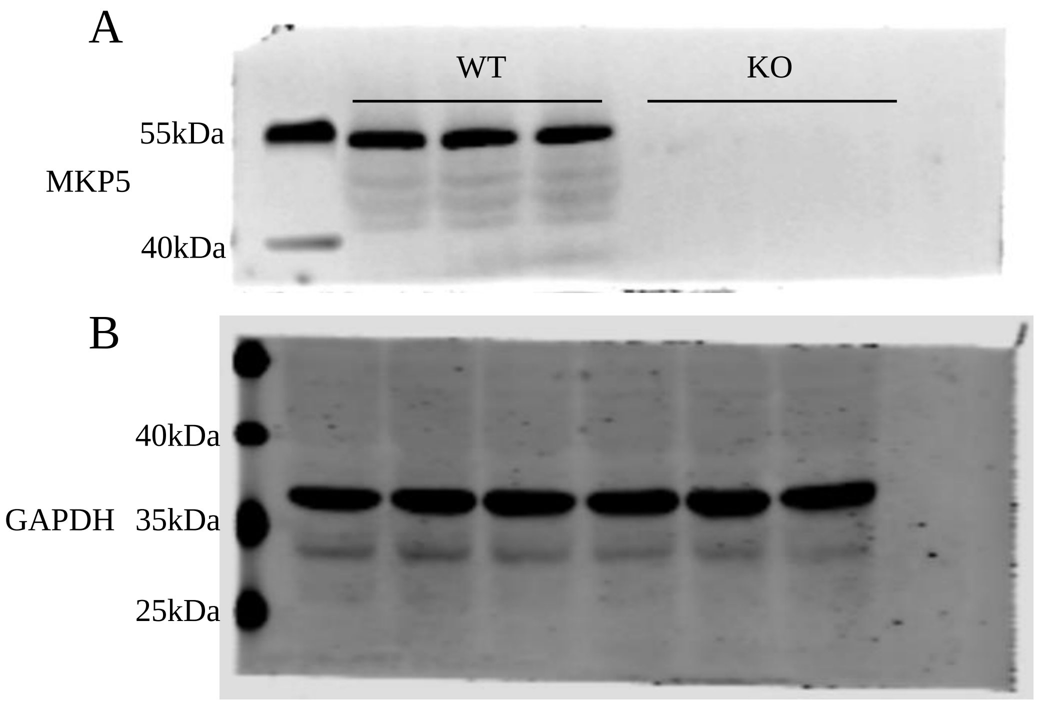


(A) MKP5 protein bands in Figure 1E; (B) GAPDH protein bands in Figure 1E.

Figure 1F

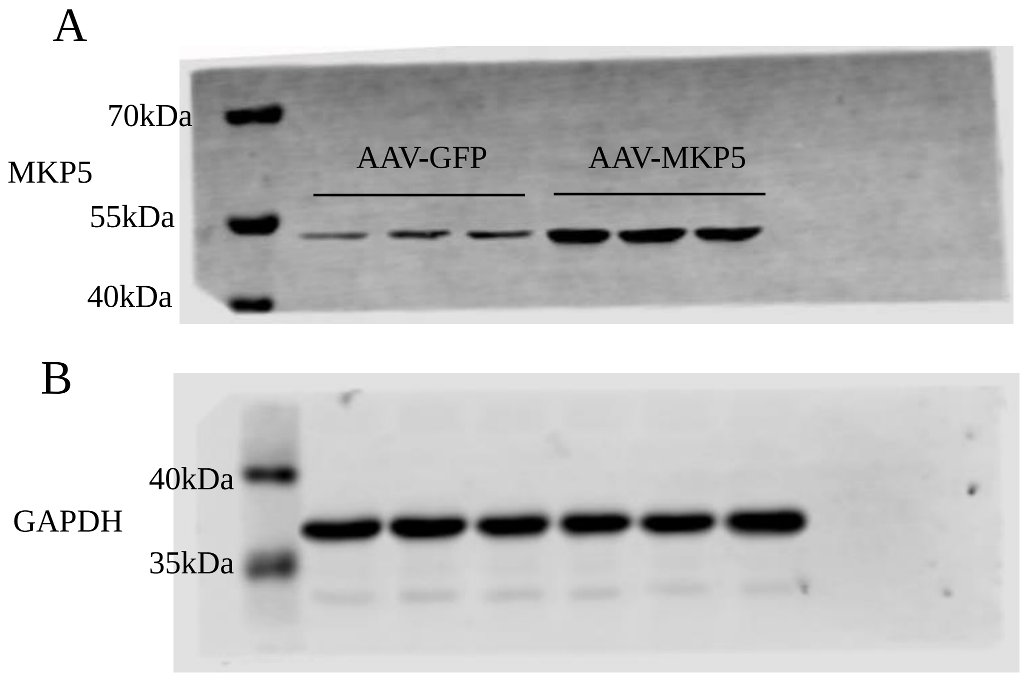


(A) MKP5 protein bands in Figure 1F; (B) GAPDH protein bands in Figure 1F.

Figure 2G

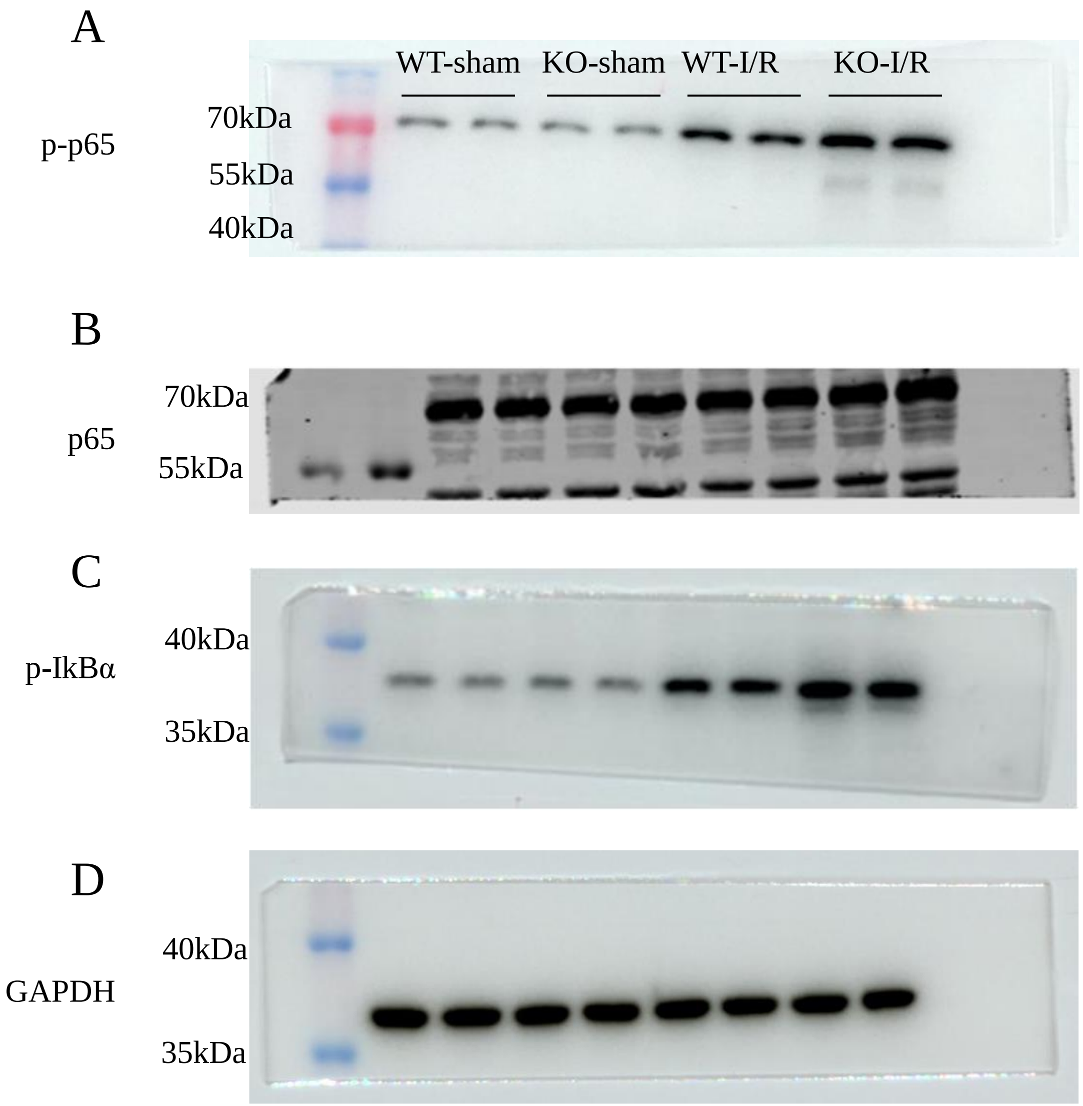


(A) p-p65 protein bands in Figure 2G; (B) p65 protein bands in Figure 2G; (C) p-IkBα protein bands in Figure 2G; (D) GAPDH protein bands in Figure 2G.

Figure 2O

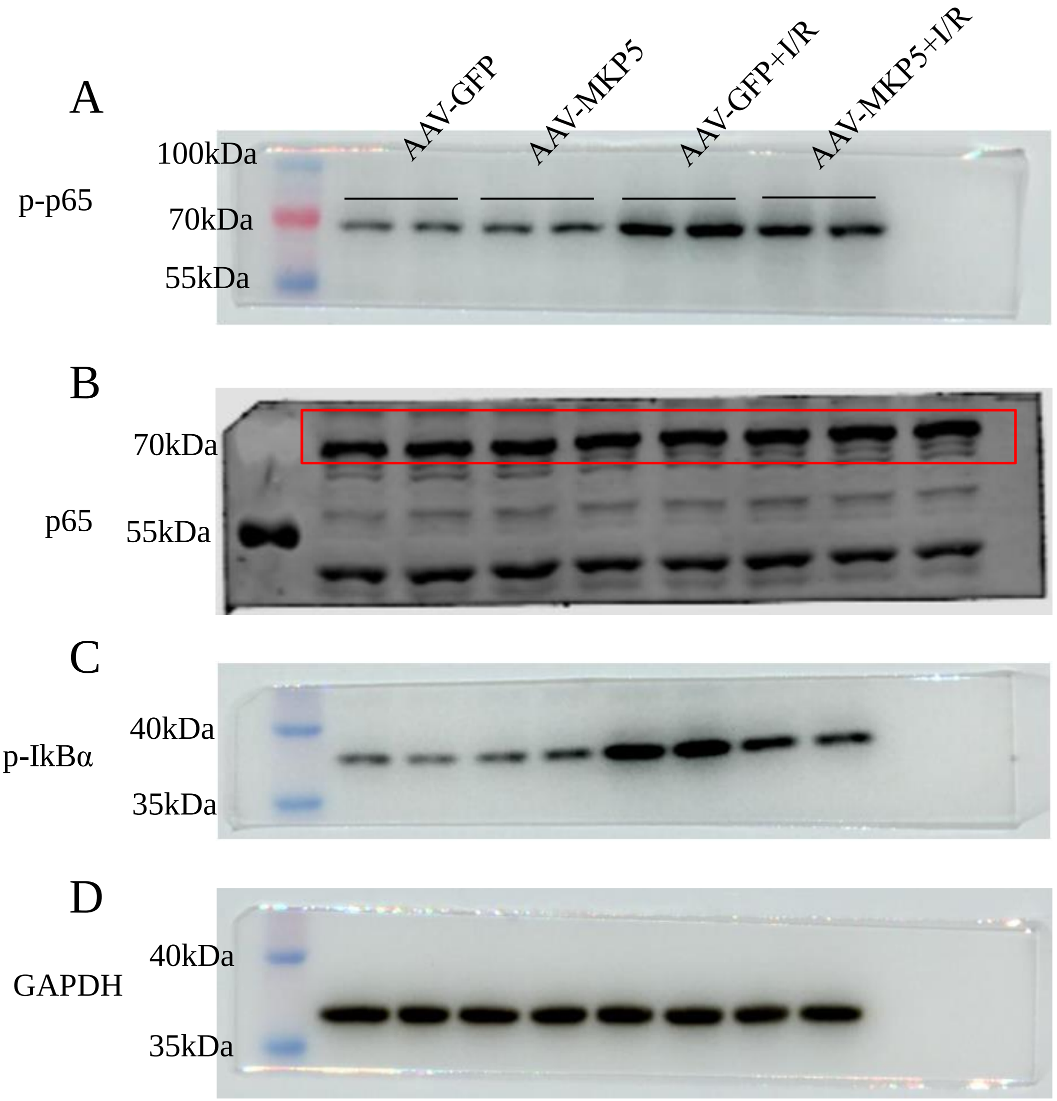


(A) p-p65 protein bands in Figure 2O; (B) p65 protein bands in Figure 2O; (C) p-IkBα protein bands in Figure 2O; (D) GAPDH protein bands in Figure 2O.

# Figure 3C

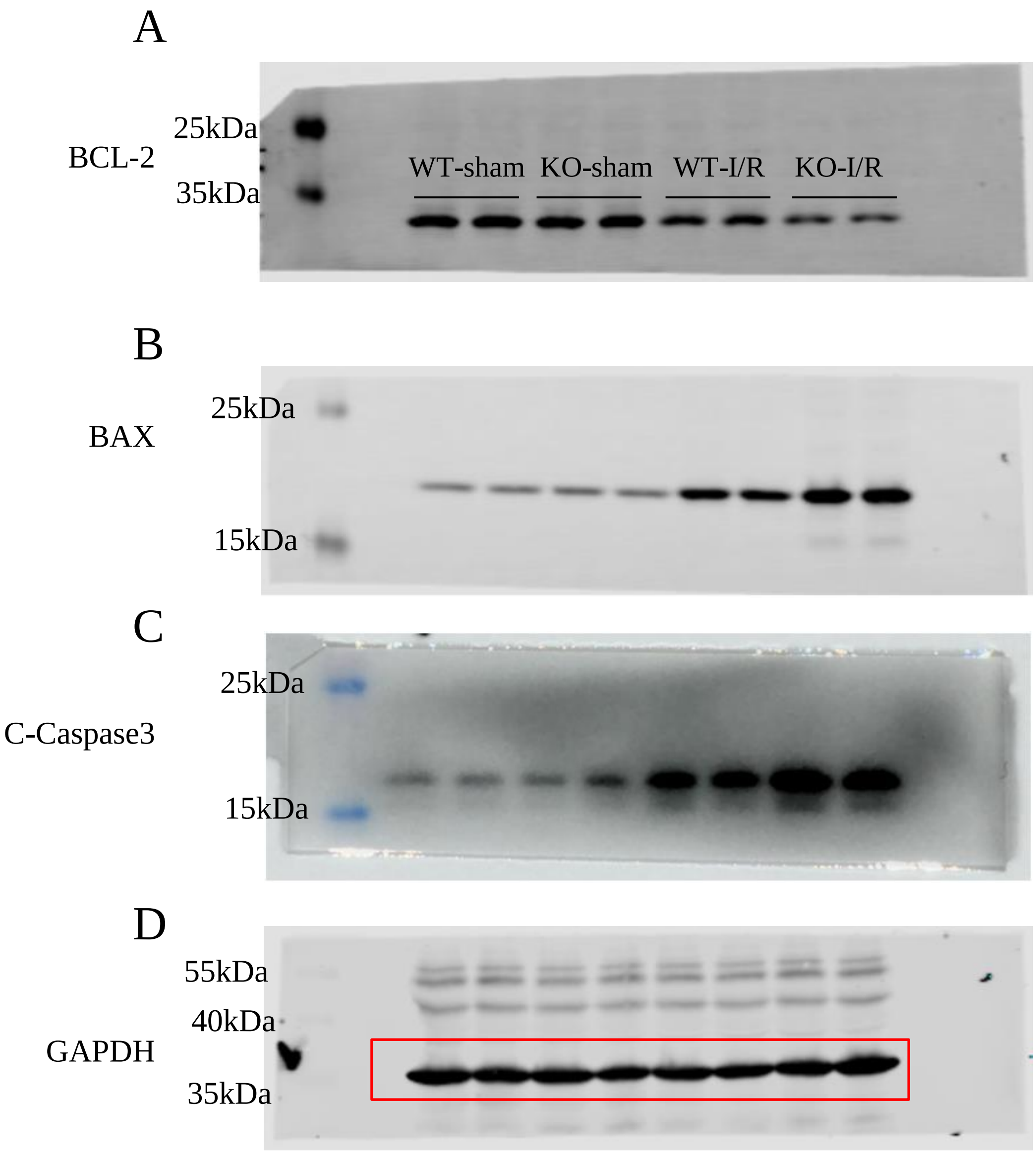


(A) BCL-2 protein bands in Figure 3C; (B) BAX protein bands in Figure 3C; (C) C-Caspase3 protein bands in Figure 3C; (D) GAPDH protein bands in Figure 3C.

Figure 3G

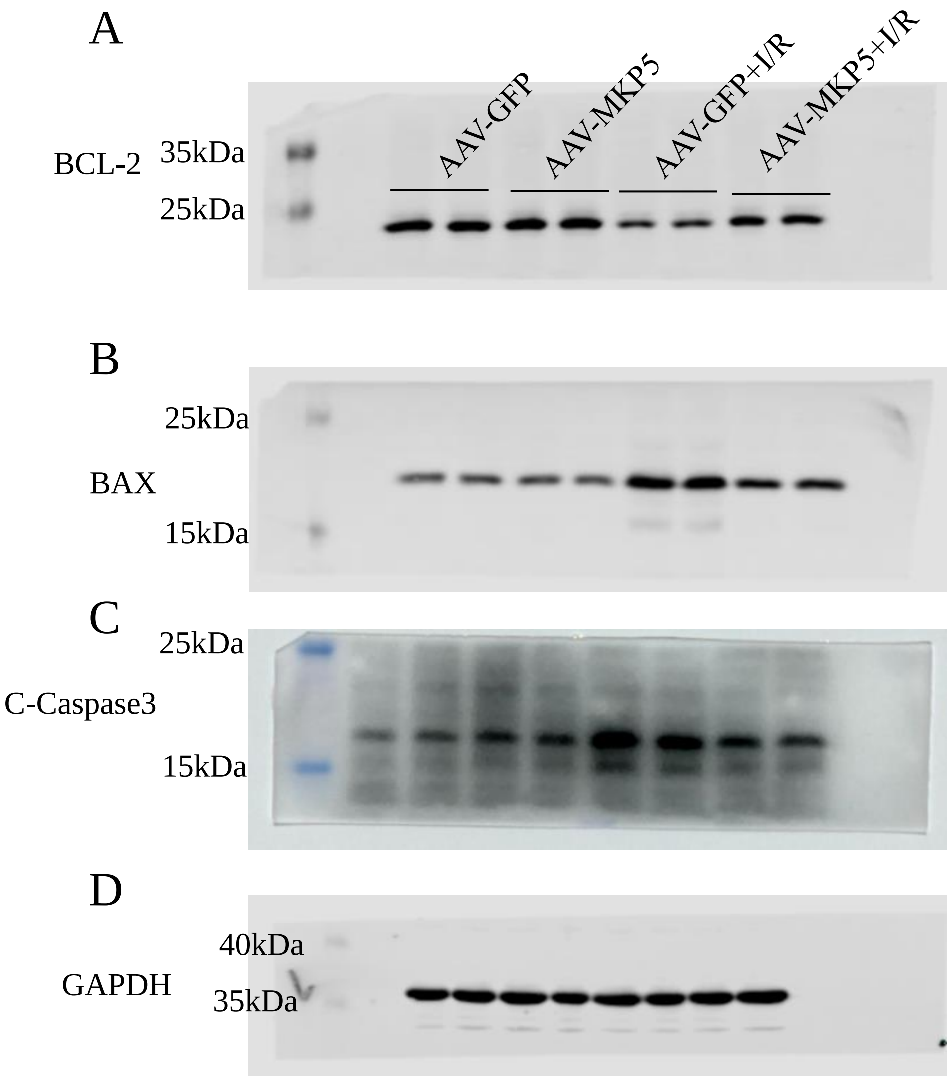


(A) BCL-2 protein bands in Figure 3G; (B) BAX protein bands in Figure 3G; (C) C-Caspase3 protein bands in Figure 3G; (D) GAPDH protein bands in Figure 3G.

Figure 5A

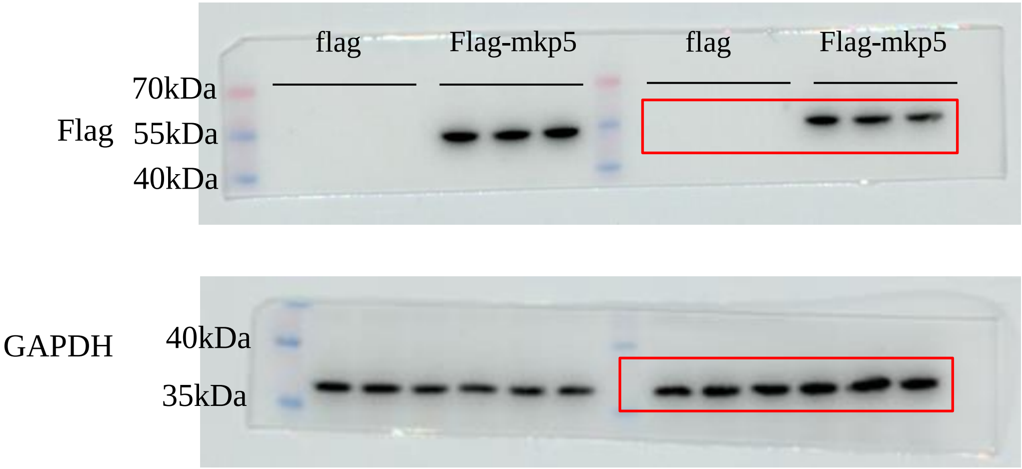


(A) Flag protein bands in Figure 5A; (B) GAPDH protein bands in Figure 5A.

Figure 5B

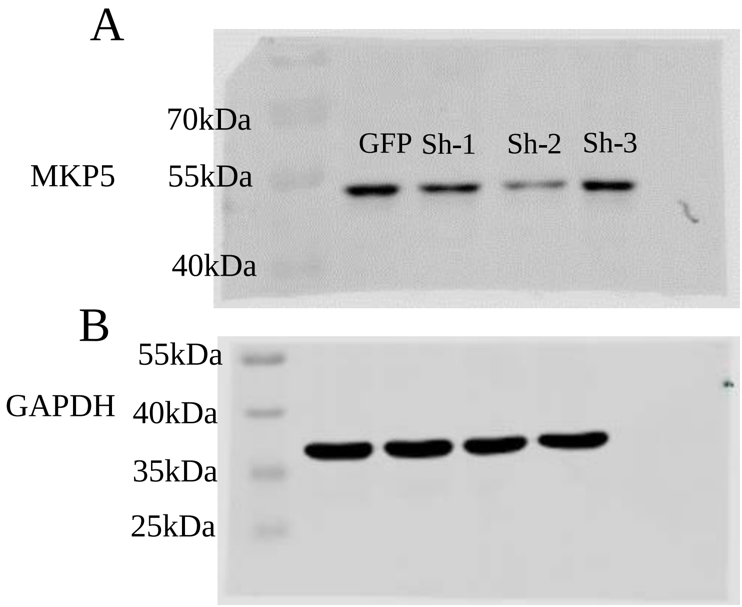


(A) MKP5 protein bands in Figure 5B; (B) GAPDH protein bands in Figure 5B.

Figure 5J

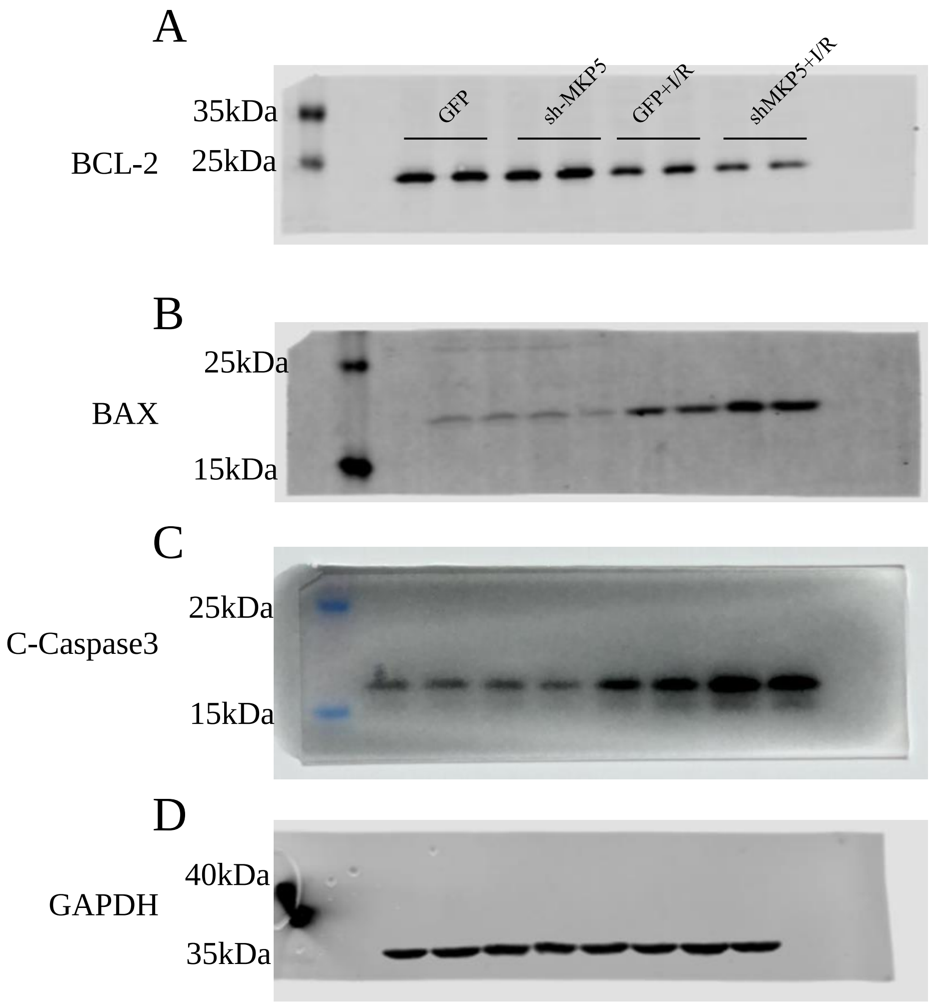


(A) BCL-2 protein bands in Figure 5J; (B) BAX protein bands in Figure 5J; (C) C-Caspase3 protein bands in Figure 5J; (D) GAPDH protein bands in Figure 5J.

Figure 5K

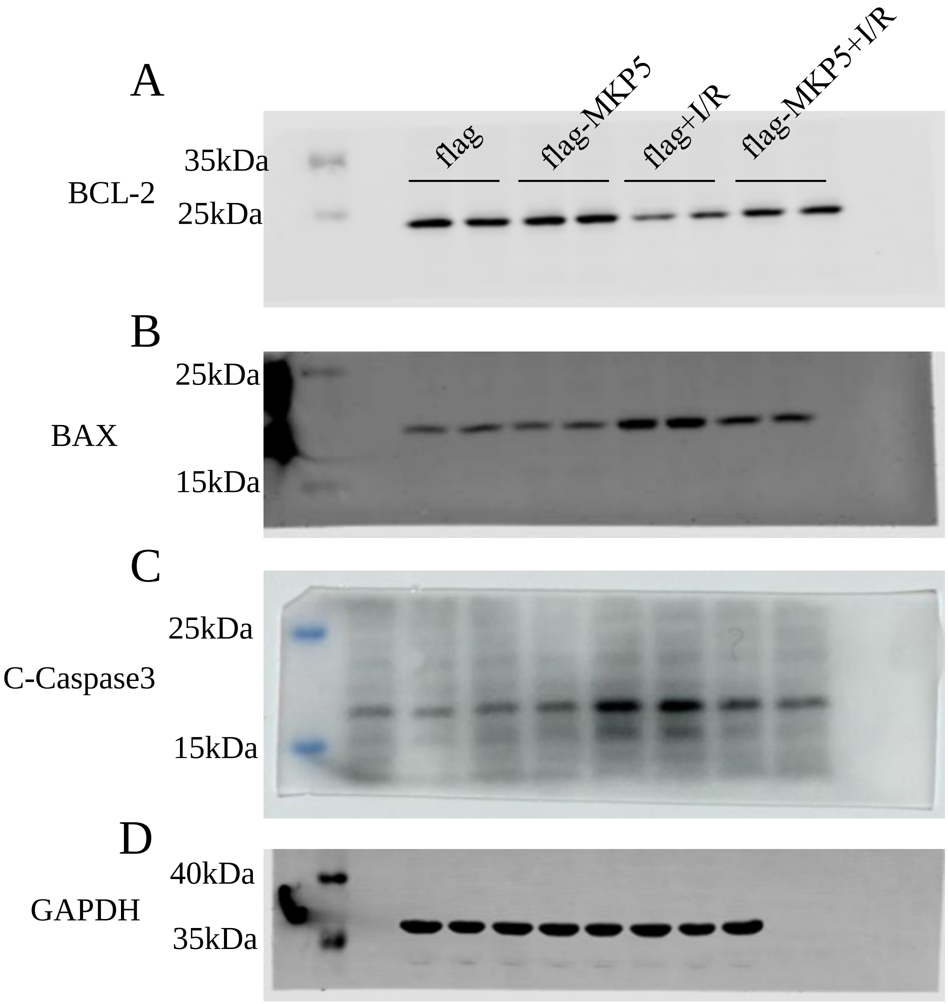


(A) BCL-2 protein bands in Figure 5N; (B) BAX protein bands in Figure 5N; (C) C-Caspase3 protein bands in Figure 5N; (D) GAPDH protein bands in Figure 5N.

Figure 6A

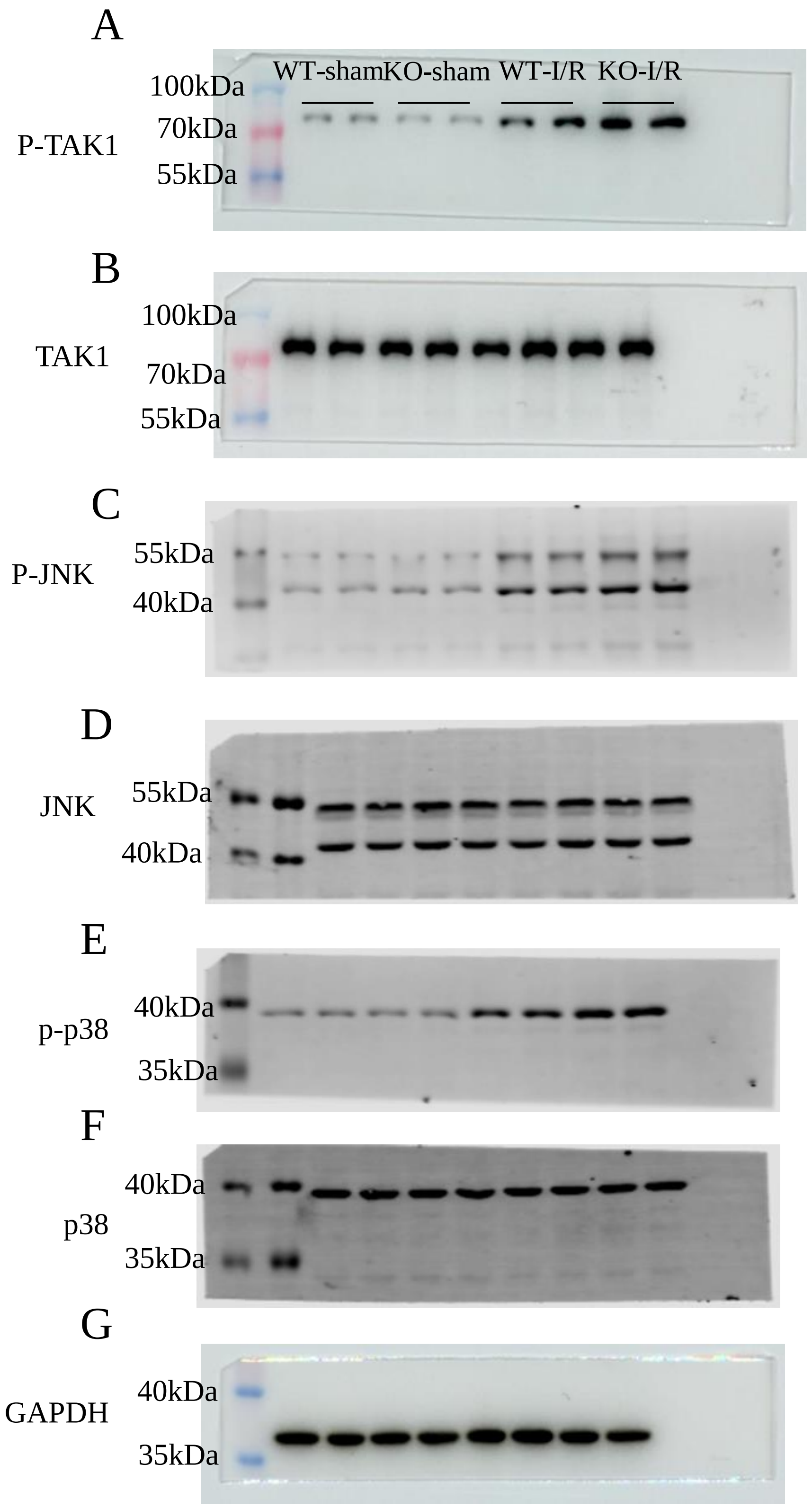


(A) p-TAK1 protein bands in Figure 6A; (B) TAK1 protein bands in Figure 6A; (C) p-JNK protein bands in Figure 6A; (D) JNK protein bands in Figure 6A; (E) p-p38 protein bands in Figure 6A; (F) p38 protein bands in Figure 6A; (G) GAPDH protein bands in Figure 6A.

Figure 6C

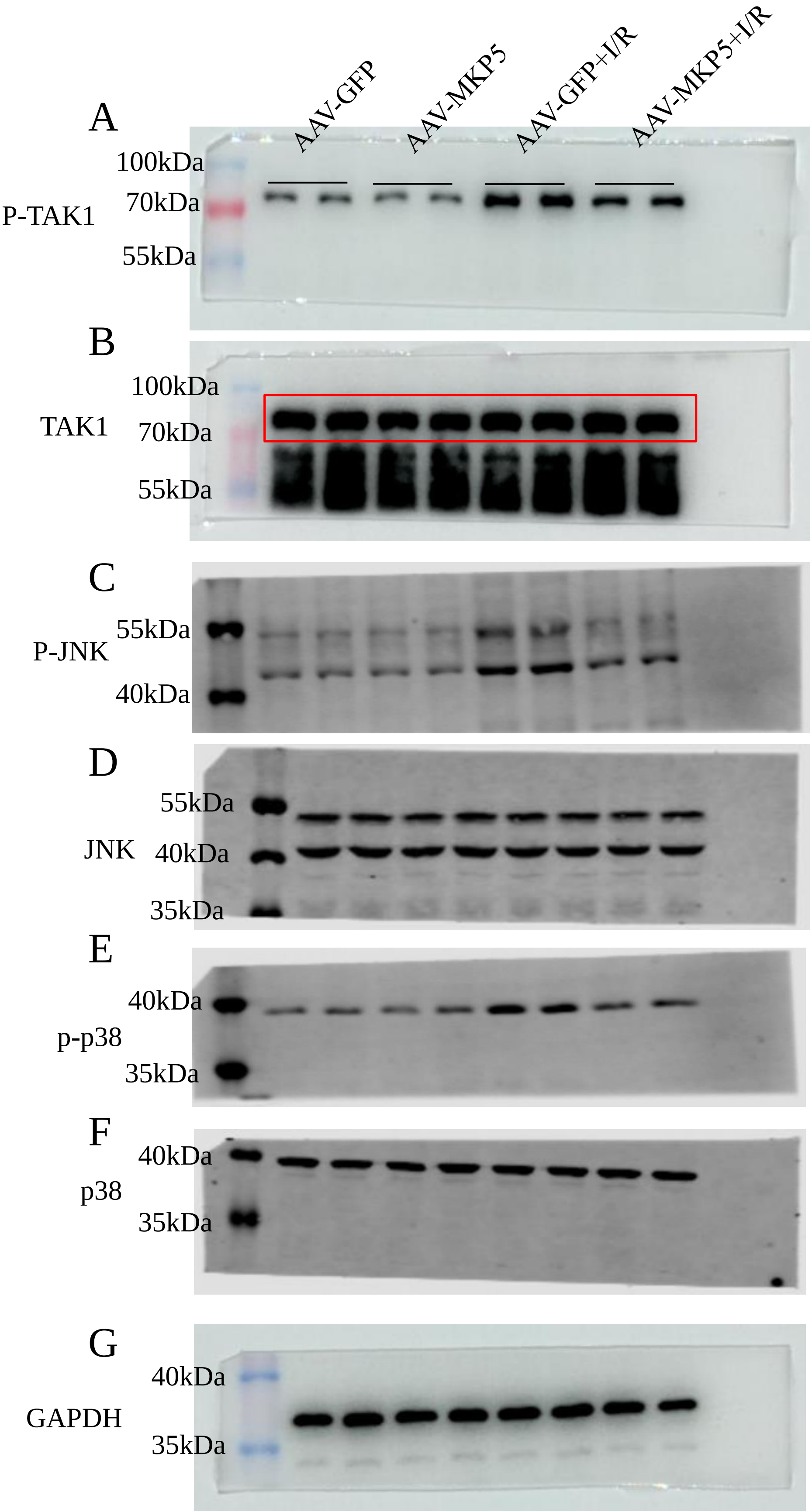


(A) p-TAK1 protein bands in Figure 6C; (B) TAK1 protein bands in Figure 6C; (C) p-JNK protein bands in Figure 6C; (D) JNK protein bands in Figure 6C; (E) p-p38 protein bands in Figure 6C; (F) p38 protein bands in Figure 6C; (G) GAPDH protein bands in Figure 6C.

Figure 6E

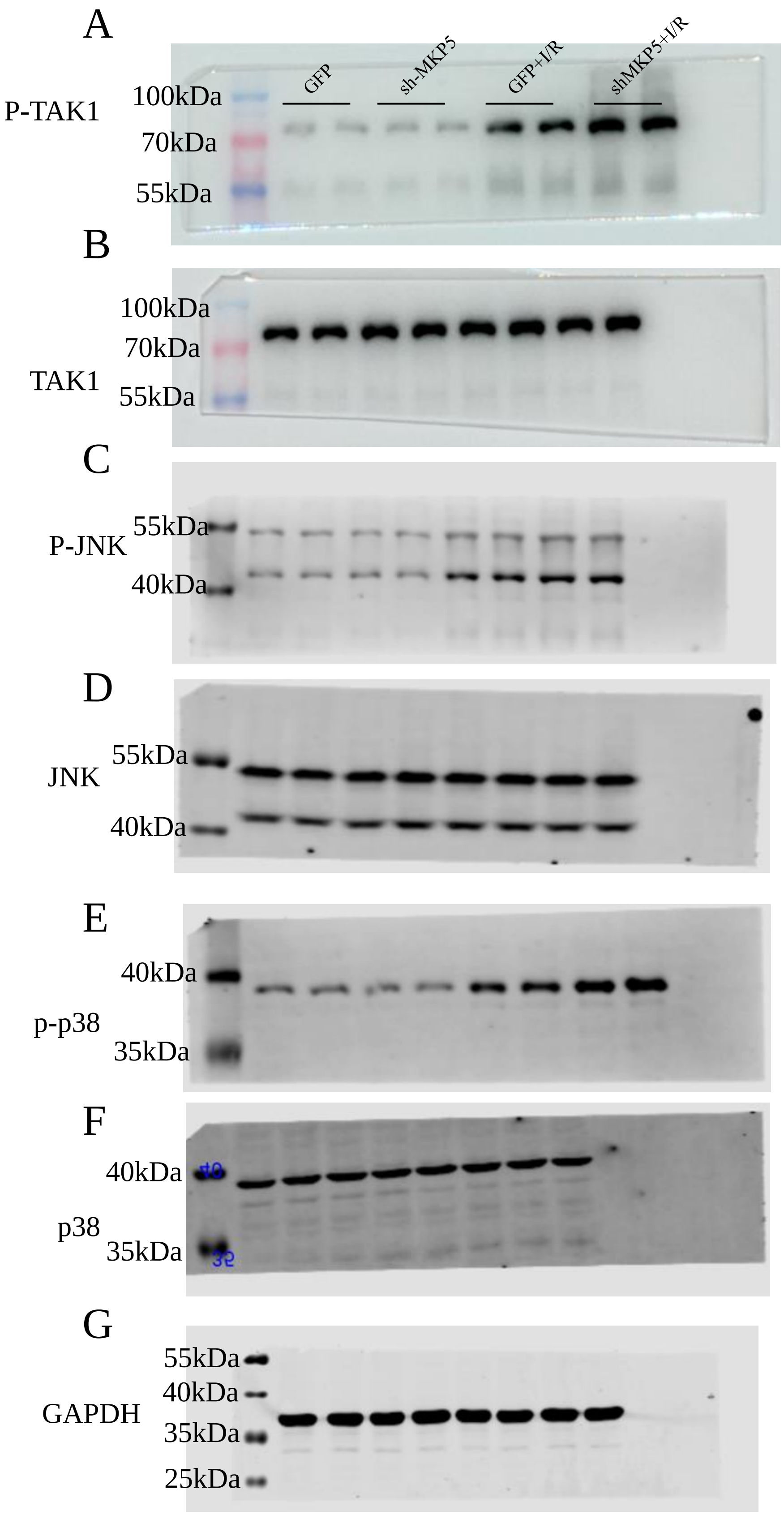


(A) p-TAK1 protein bands in Figure 6E; (B) TAK1 protein bands in Figure 6E; (C) p-JNK protein bands in Figure 6E; (D) JNK protein bands in Figure 6E; (E) p-p38 protein bands in Figure 6E; (F) p38 protein bands in Figure 6E; (G) GAPDH protein bands in Figure 6E.

Figure 6G

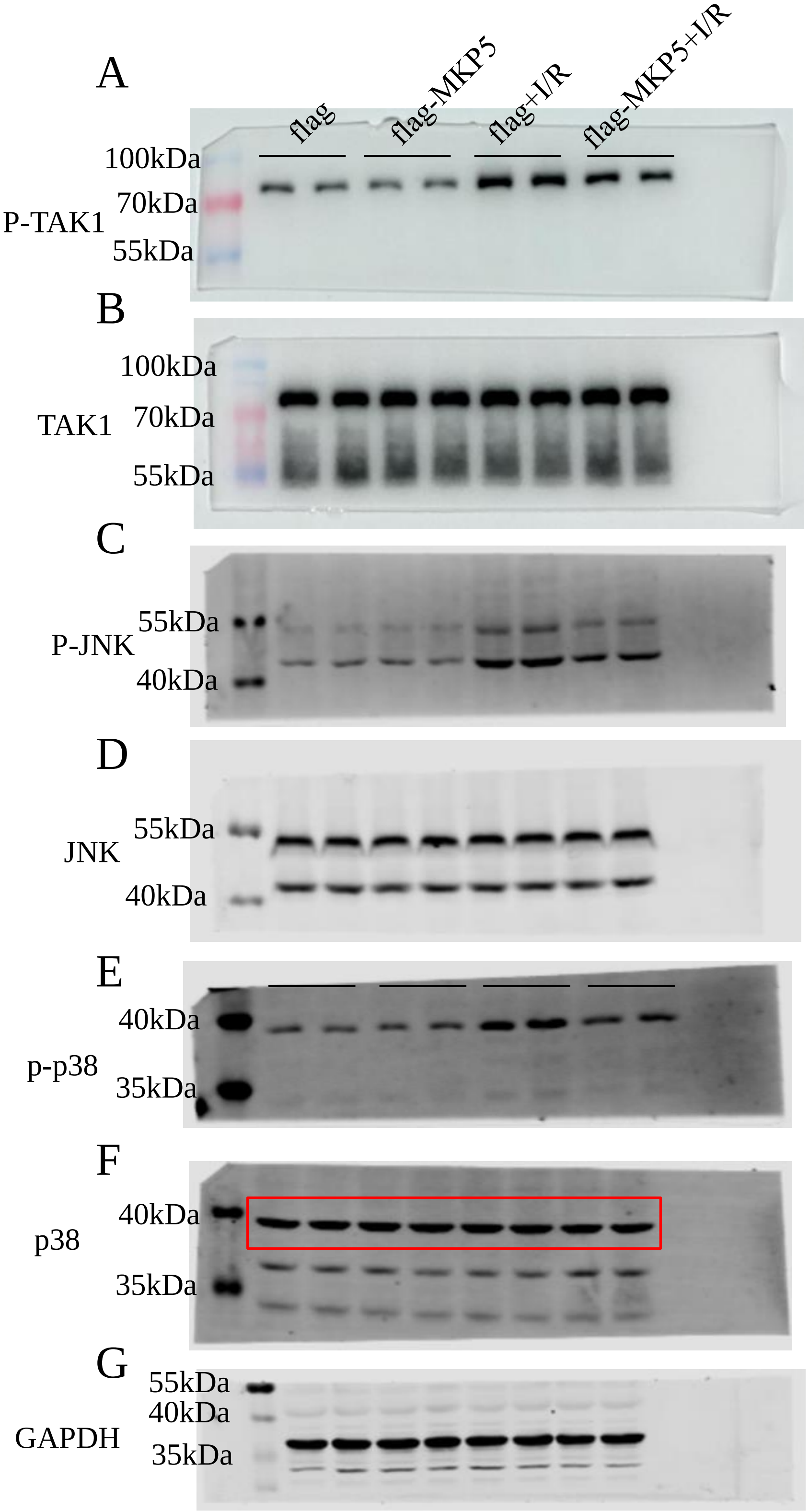


(A) p-TAK1 protein bands in Figure 6G; (B) TAK1 protein bands in Figure 6G; (C) p-JNK protein bands in Figure 6G; (D) JNK protein bands in Figure 6G; (E) p-p38 protein bands in Figure 6G; (F) p38 protein bands in Figure 6G; (G) GAPDH protein bands in Figure 6G.

Figure 7A

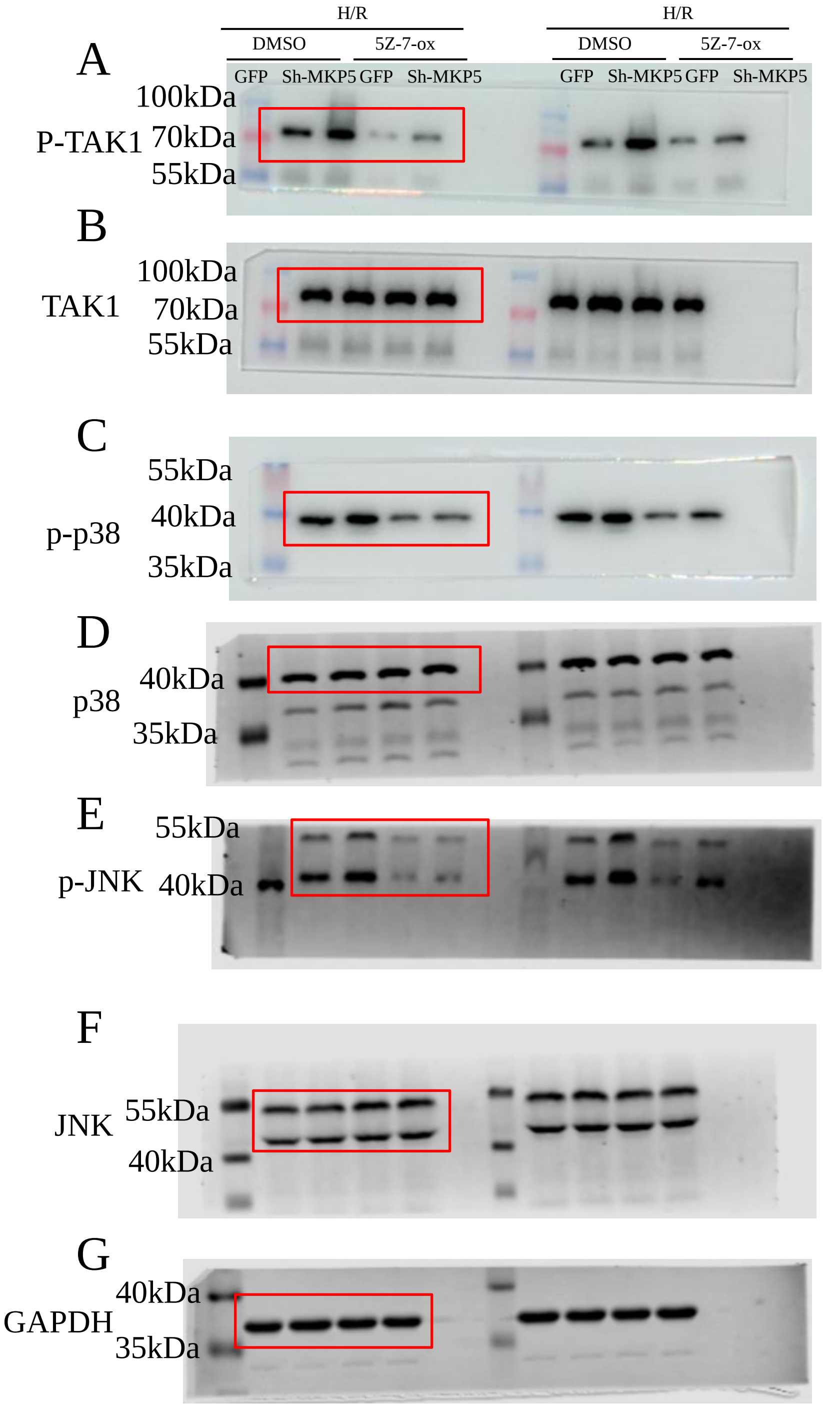


(A) p-TAK1 protein bands in Figure 7A; (B) TAK1 protein bands in Figure 7A; (C) p-p38 protein bands in Figure 7A; (D) p38 protein bands in Figure 7A; (E) p-JNK protein bands in Figure 7A; (F) JNK protein bands in Figure 7A; (G) GAPDH protein bands in Figure 7A.

Figure 7G

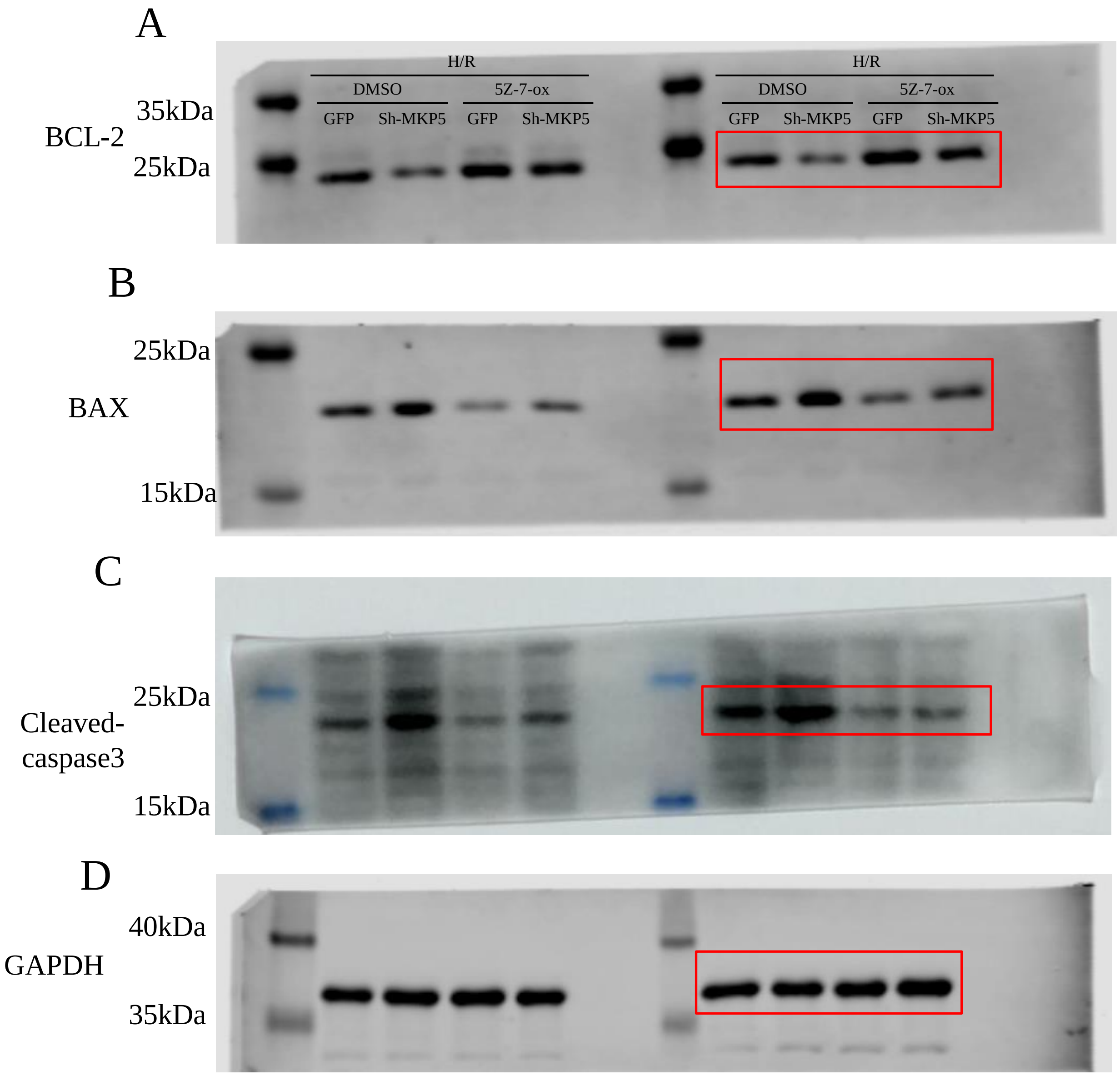


(A) BCL-2 protein bands in Figure 7G; (B) BAX protein bands in Figure 7G; (C) C-Caspase3 protein bands in Figure 7G; (D) GAPDH protein bands in Figure 7G.
